# Characterization of the first cultured free-living representative of *Candidatus* Izemoplasma uncovers its unique biology

**DOI:** 10.1038/s41396-021-00961-7

**Published:** 2021-03-21

**Authors:** Rikuan Zheng, Rui Liu, Yeqi Shan, Ruining Cai, Ge Liu, Chaomin Sun

**Affiliations:** 1 CAS and Shandong Province Key Laboratory of Experimental Marine Biology & Center of Deep Sea Research, Institute of Oceanology, Chinese Academy of Sciences, Qingdao, China; 2 Laboratory for Marine Biology and Biotechnology, Qingdao National Laboratory for Marine Science and Technology, Qingdao, China; 3 College of Earth Science, University of Chinese Academy of Sciences, Beijing, China; 4 Center of Ocean Mega-Science, Chinese Academy of Sciences, Qingdao, China

**Keywords:** Marine microbiology, Biogeochemistry

## Abstract

*Candidatus* Izemoplasma, an intermediate in the reductive evolution from Firmicutes to Mollicutes, was proposed to represent a novel class of free-living wall-less bacteria within the phylum Tenericutes. Unfortunately, the paucity of pure cultures has limited further insights into their physiological and metabolic features as well as ecological roles. Here, we report the first successful isolation of an Izemoplasma representative from the deep-sea methane seep, strain zrk13, using a DNA degradation-driven method given Izemoplasma’s prominent DNA-degradation potentials. We further present a detailed description of the physiological, genomic and metabolic traits of the novel strain, which allows for the first time the reconstruction of the metabolic potential and lifestyle of a member of the tentatively defined *Candidatus* Izemoplasma. On the basis of the description of strain zrk13, the novel species and genus *Xianfuyuplasma coldseepsis* is proposed. Using a combined biochemical and transcriptomic method, we further show the supplement of organic matter, thiosulfate or bacterial genomic DNA could evidently promote the growth of strain zrk13. In particular, strain zrk13 could degrade and utilize the extracellular DNA for growth in both laboraterial and deep-sea conditions. Moreover, the predicted genes determining DNA-degradation broadly distribute in the genomes of other Izemoplasma members. Given that extracellular DNA is a particularly crucial phosphorus as well as nitrogen and carbon source for microorganisms in the seafloor, Izemoplasma bacteria are thought to be important contributors to the biogeochemical cycling in the deep ocean.

## Introduction

The phylum Tenericutes is composed of bacteria lacking a peptidoglycan cell wall and consists of bacteria that evolved from the phylum Firmicutes [1–3]. Although some researchers strongly argued that Tenericutes should be integrated into phylum Firmicutes [2, 4], two distinctive features set Tenericutes apart from the Firmicutes: the inability to synthetize precursors of peptidoglycan and thereby forming a cell wall [5–8], and extreme reduction of the size of genome (between 530 and 2220 kbp) [9, 10]. In the current time, the taxonomic status of Tenericutes is uncertain [9]. With more novel lineages of Tenericutes bacteria are identified in the future, the taxonomic placing and monophyly of Tenericutes will probably be further challenged [2]. Nonetheless, in the time of writing, Tenericutes includes the class Mollicutes [2], three taxa in provisional class *Candidatus* Izemoplasma (the new name proposed by the Genome Taxonomy Database (GTDB)) [2, 5], and several taxa of unclassified status. Tenericutes bacteria have evolved a broad range of lifestyles, including free-living, commensalism and parasitism [2]. Up to date, almost all reported Mollicutes (including five orders Mycoplasmatales, Entomoplasmatales, Haloplasmatales, Acholeplasmatales, and Anaeroplasmatales) [11] are commensals or obligate parasites of humans, domestic animals, plants and insects [2]. In comparison, some free-living species are found to be associated with inert substrates (e.g., *Candidatus* Izemoplasma) [5] or animal/plant surfaces (e.g., *Acholeplasma laidlawii*) [12, 13].

Tenericutes bacteria ubiquitously exist in numerous environments [14]. Environmental 16S rRNA surveys have identified a large number of unknown Tenericutes clades in diverse environments including the deep oceans, introducing the possibility that these Tenericutes bacteria may represent free-living microorganisms which conducting a non-host-associated lifestyle [2]. Indeed, free-living *Candidatus* Izemoplasma [5] and Haloplasma [15] were reported in a deep-sea cold seep and brine pool, respectively. These deep-sea free-living Tenericutes exhibit metabolic versatility and adaptive flexibility, showing the possibility to isolate more Tenericutes bacteria from the oceans and even other extreme environments.

Among the free-living Tenericutes identified in the marine environments, *Candidatus* Izemoplasma was mainly studied based on the enrichment and corresponding assembled genomes given the absence of pure cultures [5, 16]. *Candidatus* Izemoplasma was proposed to represent a novel class of free-living wall-less bacteria within the phylum Tenericutes [5], and an intermediate in the reductive evolution from Firmicutes to Mollicutes [5]. Members of the Izemoplasma are thought to be heterotrophs engaging primarily in fermentative metabolism [5], and thereby producing lactate and possibly other small molecules, which in turn may be utilized by other members of the cold seep [16]. Notably, based on their genomic analysis, *Candidatus* Izemoplasma bacteria are believed to be important DNA degraders [5, 16]. Recent studies have shown high concentrations of extracellular DNA in marine sediments from shallow depths down to the abyssal floor [17–19]. It was estimated that extracellular DNA available for degradation by extracellular nucleases, supplies microbial communities of both coastal and deep-sea sediments with 2–4% of their carbon requirements, 4–7% of their nitrogen needs, and a remarkable 20–47% of their phosphorus demands [20, 21]. Though the relative abundance of *Candidatus* Izemoplasma from the marine waters was low (<0.1%) in the microbial communities of the oceans [2], considering the tremendous body of marine water, the oceans harbor a massive Izemoplasma population composed of diverse novel lineages [2]. Given extracellular DNA is a particularly crucial phosphorus as well as nitrogen and carbon source for microorganisms in the seafloor, *Candidatus* Izemoplasma might be a potential contributor to the biogeochemical cycling in the deep ocean. However, the paucity of isolates currently available has greatly limited further insights into the physiological, ecological and evolutional studies of *Candidatus* Izemoplasma.

In this study, we successfully cultivated the first free-living representative of *Candidatus* Izemoplasma. Using growth assay and transcriptomic methods, we further found organic nutrient and thiosulfate could significantly promote the growth of the new isolate. Of note, the novel isolate was found to be an effective degrader of extracellular DNA in both laboratorial and deep-sea conditions. Lastly, its metabolic pathways were reconstructed based on multi-omics results presented in this study and its potential ecological roles were also discussed.

## Materials and methods

### Samples sampling and statistical analysis of 16S rRNA genes of Tenericutes in the deep-sea cold seep

The deep-sea samples were collected by *RV KEXUE* from a typical cold seep in the South China Sea (E119°17’07.322”, N22°06’58.598”) as described previously [22]. The corresponding chemical parameters of the cold seep were shown in the Supplementary Table 1. To understand the abundance of Tenericutes in deep-sea cold seep sediments, we collected six sedimentary samples (RPC, ZC1, ZC2, ZC3, ZC4, and ZC5 at depth intervals of 0–10, 30–50, 90–110, 150–170, 210–230 and 230–250 cm, respectively) for operational taxonomic units (OTUs) sequencing that performed by Novogene (Tianjin, China). Total DNAs from these samples were extracted by the CTAB/SDS method [23] and were diluted to 1 ng/µL with sterile water and used for PCR template. 16S rRNA genes of distinct regions (16S V3/V4) were amplified using specific primers (341F: 5′- CCTAYGGGRBGCASCAG and 806R: 5′- GGACTACNNGGGTATCTAAT) with the barcode. These PCR products were purified with a Qiagen Gel Extraction Kit (Qiagen, Germany) and prepared to construct libraries. Sequencing libraries were generated using TruSeq® DNA PCR-Free Sample Preparation Kit (Illumina, USA) following the manufacturer’s instructions. The library quality was assessed on the Qubit@ 2.0 Fluorometer (Thermo Scientific) and Agilent Bioanalyzer 2100 system. And then the library was sequenced on an Illumina NovaSeq platform and 250 bp paired-end reads were generated. Paired-end reads were merged using FLASH (V1.2.7, http://ccb.jhu.edu/software/FLASH/) [24], which was designed to merge paired-end reads when at least some of the reads overlap those generated from the opposite end of the same DNA fragments, and the splicing sequences were called raw tags. Quality filtering on the raw tags were performed under specific filtering conditions to obtain the high-quality clean tags [25] according to the QIIME (V1.9.1, http://qiime.org/scripts/split_libraries_fastq.html) quality controlled process. The tags were compared with the reference database (Silva database, release 111-2012_07, https://www.arb-silva.de/) using UCHIME algorithm (UCHIME Algorithm, http://www.drive5.com/usearch/manual/uchime_algo.html) [26] to detect chimera sequences, and then the chimera sequences were removed [27]. And sequence analyses were performed by Uparse software (Uparse v7.0.1001, http://drive5.com/uparse/) [28]. Sequences with ≥97% similarity were assigned to the same OTUs. The representative sequence for each OTU was screened for further annotation. For each representative sequence, the Silva Database (release 111-2012_07, http://www.arb-silva.de/) [29] was used based on Mothur algorithm to annotate taxonomic information.

### Metagenomic sequencing, assembly, binning and annotation

We selected three cold seep sediment samples (ZC1, ZC3 and ZC4, 20 g each) for metagenomic analysis in BGI (BGI, China). Briefly, total DNAs from these samples were extracted using the a Qiagen DNeasy® PowerSoil® Pro Kit (Qiagen, Germany) and the integrity of DNA was evaluated by agarose gel electrophoresis. 0.5 μg DNA of each sample was used for libraries preparation with an amplification step. Thereafter, DNA was cleaved into 50–800 bp fragments using Covaris E220 ultrasonicator (Covaris, UK) and some fragments between 150–250 bp were selected using AMPure XP beads (Agencourt, USA) and repaired using T4 DNA polymerase (ENZYMATICS, USA). All NGS sequencing was performed on the BGISEQ-500 platform (BGI, China), generating 100 bp paired-end raw reads. Quality control was performed by SOAPnuke (v1.5.6) (setting: -l 20 -q 0.2 -n 0.05 -Q 2 -d -c 0 -5 0 -7 1) [30] and the clean data were assembled using MEGAHIT (v1.1.3) (setting:--min-count 2 --k-min 33 --k-max 83 --k-step 10) [31]. Maxbin2 [32], metaBAT2 [33] and Concoct[34] were used to automatically bin from assemblies of these samples. MetaWRAP [35] was used to purify and organize data to generate the final bins. Finally, completeness and contamination of metagenome-assembled genomes (MAGs) were assessed using checkM (v1.0.18) [36]. These obtained MAGs were subsequently annotated by searching the predicted genes against NR (20180814), KEGG (Release 87.0), COG (update-2018_08) and Swissprot (release-2017_07).

### Isolation and cultivation of deep-sea Izemoplasma

To enrich the Izemoplasma bacteria, the sediment samples were cultured at 28 °C for 6 months in an anaerobic basal medium supplemented with 1.0 mg/L *Escherichia coli* genomic DNA. The compositions of basal medium are 1.0 g NH_4_Cl, 1.0 g NaHCO_3_, 1.0 g CH_3_COONa, 0.5 g KH_2_PO_4_, 0.2 g MgSO_4_^.^7H_2_O, 0.7 g cysteine hydrochloride, 500 µL 0.1 % (w/v) resazurin in 1 liter seawater, pH 7.0. This medium was prepared anaerobically as previously described [37]. During the course of enrichment, 1.0 mg *E. coli* genomic DNA was added once a month. After a 6-month enrichment, 50 µL dilution portion was spread on Hungate tube covered by the enrichment medium supplemented with 15.0 g/L agar. The Hungate tubes were anaerobically incubated at 28 °C for 7 days. Individual colonies with distinct morphology were picked using sterilized bamboo sticks and then cultured in the basal medium. Strain zrk13 was isolated and purified with basal medium by repeated use of the Hungate roll-tube methods for several rounds until it was considered to be axenic. The purity of strain zrk13 was confirmed routinely by TEM and repeated partial sequencing of the 16S rRNA gene. Strain zrk13 is preserved at −80 °C in the basal medium supplemented with 20% (v/v) glycerol. Since strain zrk13 grew slowly in the basal medium, we added extra organic substances in the medium as following: 1.0 g/L yeast extract, 1.0 g/L peptone, 1.0 g/L NH_4_Cl, 1.0 g/L NaHCO_3_, 1.0 g/L CH_3_COONa, 0.5 g/L KH_2_PO_4_, 0.7 g/L cysteine hydrochloride, 500 µL/L of 0.1 % (w/v) resazurin, 1 L seawater and pH 7.0, and the corresponding medium was named ‘rich medium’ in this study.

The temperature, pH and NaCl concentration ranges for the growth of strain zrk13 were determined in the rich medium incubated for 14 days. Growth assays were performed at different temperatures (4, 16, 24, 28, 32, 37, 45, 60, 70, 80 °C). The pH range for growth was tested from pH 2.0 to pH 10.0 with increments of 0.5 pH units. Salt tolerance was tested in the modified rich medium (dissolved in distilled water) supplemented with 0–10% (w/v) NaCl (1.0% intervals). Substrates utilization of strain zrk13 was tested in the medium (pH 7.0) consisting of (L^−1^): 5 g NaCl, 1 g NH_4_Cl, 0.5 g KH_2_PO4, 0.2 g MgSO_4_, 0.02 g yeast extract. Single substrate (including glucose, maltose, butyrate, fructose, sucrose, acetate, formate, starch, isomaltose, trehalose, galactose, cellulose, xylose, lactate, ethanol, D-mannose, glycerin, rhamnose, and sorbitolurea) was added from sterile filtered stock solutions to the final concentration at 20 mM, respectively. Cell culture containing only 0.02 g yeast extract (L^−1^) without adding any other substrates was used as a control. All the cultures were incubated at 28 °C for 14 days. For each substrate, three biological replicates were performed.

### TEM observation

To observe the morphological characteristics of zrk13, its cell suspension was washed with Milli-Q water and centrifuged at 5,000 × *g* for 5 min, and taken by immersing copper grids coated with a carbon film for 20 min, washed for 10 min in distilled water and dried for 20 min at room temperature [38]. Ultrathin-section electron microscopic observation was performed as described previously [39, 40]. Briefly, the samples were firstly preserved in 2.5% (v/v) glutaraldehyde overnight at 4 °C, washed three times with PBS and dehydrated in ethanol solutions of 30%, 50%, 70%, 90% and 100% for 10 min each time, and then the samples were embedded in a plastic resin. Ultrathin sections (50–70 nm) of cells were prepared with an ultramicrotome (Leica EM UC7), stained with uranyl acetate and lead citrate. All samples were examined using TEM (HT7700, Hitachi, Japan) with a JEOL JEM 12000 EX (equipped with a field emission gun) at 100 kV.

### Genome sequencing and genomic characteristics of strain zrk13

Genomic DNA was extracted from strain zrk13 cultured for 6 days at 28 °C. The DNA library was prepared using the Ligation Sequencing Kit (SQK-LSK109), and sequenced using a FLO-MIN106 vR9.4 flow-cell for 48 h on MinKNOWN software v1.4.2 (Oxford Nanopore Technologies (ONT), United Kingdom). Whole-genome sequence determinations of strain zrk13 were carried out with the Oxford Nanopore MinION (Oxford, United Kingdom) and Illumina MiSeq sequencing platform (San Diego, CA). A hybrid approach was utilized for genome assembly using reads from both platforms. Base-calling was performed using Albacore software v2.1.10 (ONT). Nanopore reads were processed using protocols toolkit for quality control and downstream analysis [41]. Filtered reads were assembled using Canu version 1.8 [42] using the default parameters for Nanopore data. And then the genome was assembled into a single contig and was manually circularized by deleting an overlapping end.

The genome relatedness values were calculated by multiple approaches: Average Nucleotide Identity (ANI) based on the MUMMER ultra-rapid aligning tool (ANIm), ANI based on the BLASTN algorithm (ANIb), the tetranucleotide signatures (Tetra), and in silico DNA-DNA similarity. ANIm, ANIb, and Tetra frequencies were calculated using JSpecies WS (http://jspecies.ribohost.com/jspeciesws/). The recommended species criterion cut-offs were used: 95% for the ANIb and ANIm and 0.99 for the Tetra signature. The amino acid identity (AAI) values were calculated by AAI-profiler (http://ekhidna2.biocenter.helsinki.fi/AAI/). The in silico DNA-DNA similarity values were calculated by the Genome-to-Genome Distance Calculator (GGDC) (http://ggdc.dsmz.de/) [43]. The *is*DDH results were based on the recommended formula 2, which is independent of genome size and, thus, is robust when using whole-genome sequences. The prediction of genes involved in DNA degradation for individual genomes was performed using Galaxy (Galaxy Version 2.6.0, https://galaxy.pasteur.fr/) [44] with the NCBI BLAST + blastp method.

### Phylogenetic analysis

The 16S rRNA gene tree was constructed with the full-length 16S rRNA sequences by the neighbor-joining algorithm, maximum likelihood and minimum-evolution methods. The full-length 16S rRNA gene sequence (1537 bp) of strain zrk13 was obtained from the genome (accession number MW132883), and other related taxa used for phylogenetic analysis were obtained from NCBI (www.ncbi.nlm.nih.gov/). A genome-based phylogenetic tree was reconstructed using the GTDB Toolkit (https://github.com/Ecogenomics/GTDBTk) based on the concatenated alignment of 120 ubiquitous single-copy proteins [4]. Phylogenetic trees were constructed using W-IQ-TREE web server (http://iqtree.cibiv.univie.ac.at) [45] with LG + F + I + G4 model. The online tool Interactive Tree of Life (iTOL v4) [46] was used for editing the tree.

### Growth assay of *X*. *coldseepsis* zrk13

Growth assays were performed at atmospheric pressure. In order to detect the effect of nutrients on the growth of strain zrk13, the rich medium and oligotrophic medium (containing 0.1 g/L yeast extract, 0.1 g/L peptone, 1.0 g/L NH_4_Cl, 1.0 g/L NaHCO_3_, 1.0 g/L CH_3_COONa, 0.5 g/L KH_2_PO_4_, 0.2 g/L MgSO_4_^.^7H_2_O, 0.7 g/L cysteine hydrochloride, 500 µL/L of 0.1 % (w/v) resazurin, 1 L seawater, pH 7.0) were used for assays. To detect the effects of different sulfur sources (Na_2_SO_4_ and Na_2_S_2_O_3_ at 20, 40, 60, 100 and 200 mM, Na_2_SO_3_ and Na_2_S at 0.5, 1, 2, 4 and 8 mM, respectively) on the growth of strain zrk13, the modified rich medium (containing 1.0 g/L NH_4_Cl, 1.0 g/L NaHCO_3_, 1.0 g/L CH_3_COONa, 0.5 g/L KH_2_PO_4_, 1.0 g/L yeast extract, 1.0 g/L peptone, 10 g/L NaCl, 0.7 g/L cysteine hydrochloride, 500 µL/L of 0.1% (w/v) resazurin, 1 L distilled water, pH 7.0) was used. For detecting the effect of extracellular DNA on the growth of strain zrk13, the rich medium and the basal medium (containing 1.0 g/L NH_4_Cl, 1.0 g/L NaHCO_3_, 1.0 g/L CH_3_COONa, 0.5 g/L KH_2_PO_4_, 0.2 g/L MgSO_4_^.^7H_2_O, 0.7 g/L cysteine hydrochloride, 500 µL/L of 0.1% (w/v) resazurin, 1 L seawater, pH 7.0) supplemented with or without 1.0 mg/L *E. coli* genomic DNA were used. For all the growth assays, 16 mL strain zrk13 culture was inoculated in 2 L Hungate bottles containing 1.6 L different mediums. All of the Hungate bottles were anaerobically incubated at 28 °C. Bacterial growth status was monitored by measuring the OD_600_ value via a microplate reader (Infinite M1000 Pro; Tecan, Mannedorf, Switzerland) every day until cell growth reached the stationary phase. Since strain zrk13 grew very slow in the basal medium, we also adopted the quantitative PCR (qPCR) to measure its growth status.

### Detection of DNA degradation by strain zrk13

Agarose gel electrophoresis was performed to detect the ability of DNA degradation of strain zrk13. Briefly, the *E. coli* genomic DNA/RNA was extracted from overnight cultured *E. coli* DH5α cells with a Genomic DNA and RNA Kit (Tsingke, China) by omitting the RNA removal step. The reaction system (20 µL) contained 2 µL bacterial supernatant, 2 µL FastDigest Green Buffer (10×) (Thermo Fisher Scientific, USA) and 16 µL *E. coli* genomic DNA/RNA extracts (2 µg). In parallel, the medium without cells and the supernatant of a Clostridial bacterium were used as the control groups. All bacterial supernatants were obtained by centrifugation with 12,000 *g* for 10 min from 10-day cultures. Assays were performed at 4 °C for 6 h and 12 h, or 37 °C for 5 min, 10 min, 15 min. Finally, the existence and concentration of genomic DNA/RNA in the reaction solutions were respectively checked by 1% agarose gel electrophoresis at 180 V for 20 min and Nanordrop (IMPLEN, Gemany). The imaging of the gel was taken by the Gel Image System (Tanon 2500, China).

### Quantitative real-time PCR assay

For qRT-PCR, cells of strain zrk13 were cultured in the basal medium supplemented with or without 1.0 mg/L *E. coli* genomic DNA or rich medium at 28 °C for 15 days, and 100 mL cell culture was collected from different media every day. Total RNAs from each sample were extracted using the Trizol reagent (Solarbio, China) and the RNA concentration was measured using Qubit® RNA Assay Kit in Qubit® 2.0 Flurometer (Life Technologies, CA, USA). Then RNAs from corresponding sample were reverse transcribed into cDNA and the transcriptional levels of different genes were determined by qRT-PCR using SybrGreen Premix Low rox (MDbio, China) and the QuantStudioTM 6 Flex (Thermo Fisher Scientific, USA). The PCR condition was set as following: initial denaturation at 95 °C for 3 min, followed by 40 cycles of denaturation at 95 °C for 10 s, annealing at 60 °C for 30 s, and extension at 72 °C for 30 s. 16S rRNA was used as an internal reference and the gene expression was calculated using the 2^−ΔΔCt^ method, with each transcript signal normalized to that of 16S rRNA. Transcript signals for each treatment were compared to those of control group. Specific primers for genes related to DNA degradation and 16S rRNA were designed using Primer 5.0 as shown in Supplementary Table 2. The standard curve was generated using five independent dilution series of plasmid DNA carrying a fragment (143 bp) of the strain zrk13^T^ 16S rRNA gene. This qPCR assay standard curve had a slope of −2.5162, a y-intercept of 30.53 and an *R*^2^ value of 0.9924. All qRT-PCR runs were performed in three biological and three technical replicates.

### Transcriptomic analysis

Transcriptomic analysis was performed by Novogene (Tianjin, China). Briefly, cells suspension of *X*. *coldseepsis* zrk13 cultured in the oligotrophic medium, rich medium, and modified rich medium supplemented with or without 100 mM Na_2_S_2_O_3_ for 7 days was respectively collected for further transcriptomic analysis. All cultures were performed in 2 L anaerobic bottles. For in situ experiments, *X*. *coldseepsis* zrk13 was firstly cultured in the rich medium for 5 days, and then was divided into two parts: one part was divided into three gas samples bags (which not allowing any exchanges between inside and outside; Aluminum-plastic composite film, Hede, China) and used as the control group; the other part was divided into three dialysis bags (8000–14,000 Da cutoff, which allowing the exchanges of substances smaller than 8000 Da but preventing bacterial cells from entering or leaving the bag; Solarbio, China) and used as the experimental group. All the samples were placed simultaneously in the deep-sea cold seep (E119°17′04.429″, N22°07′01.523″) for 10 days in the June of 2020 during the cruise of *Kexue* vessel. After 10 days incubation in the deep sea, the bags were taken out and the cells were immediately collected and saved in the −80 °C freezer until further use. Thereafter, the cells were checked by 16S rRNA sequencing to confirm the purity of the culture and performed further transcriptomic analyses. For transcriptomic analyses, total *X*. *coldseepsis* zrk13 RNAs were extracted using TRIzol reagent (Invitrogen, USA) and DNA contamination was removed using the MEGA clear^™^ Kit (Life technologies, USA). Detailed protocols of the following procedures including library preparation, clustering and sequencing and data analyses were described in the Supplementary information.

## Results

### Quantification of the abundance of Tenericutes and *Candidatus* Izemoplasma in the deep-sea cold seep sediments

To gain some preliminary understanding of Tenericutes in the deep sea, OTUs sequencing was performed to detect the abundance of Tenericutes present in the cold seep sediments at depth intervals of 0–10 cm (sample RPC), 30–50 cm (sample ZC1), 90–110 cm (sample ZC2), 150–170 cm (sample ZC3), 210–230 cm (sample ZC4), 230–250 cm (sample ZC5) from the surface to the deep layer. As previously reported [2], the phylum Tenericutes had a very low abundance in the cold seep sediments (Supplementary Fig. 1A). For example, the percentages of Tenericutes only respectively accounted for 0.011%, 0.222% and 0.003% of the whole bacterial domain in the samples ZC1, ZC3 and ZC4 (Supplementary Fig. 1A). We even could not detect any Tenericutes in other three samples. To obtain further insights into the Tenericutes in deep-sea sediments, we performed metagenomics sequencing of the sample ZC3 and analyzed all the annotated genes. The results showed that sequences associated with phylum Tenericutes represented 0.250% of all annotated bacterial sequences (Supplementary Fig. 1B), which was consistent with the OTUs result of the sample ZC3 (Supplementary Fig. 1A). Of note, the proportion of genes associated with *Candidatus* Izemoplasma to those related to Tenericutes was up to 93.289% (Supplementary Fig. 1C), indicating the dominant status of *Candidatus* Izemoplasma within the deep-sea Tenericutes.

### DNA degradation-driven isolation of the first cultured free-living representative of *Candidatus* Izemoplasma from the deep-sea cold seep

**Fig. 1:**
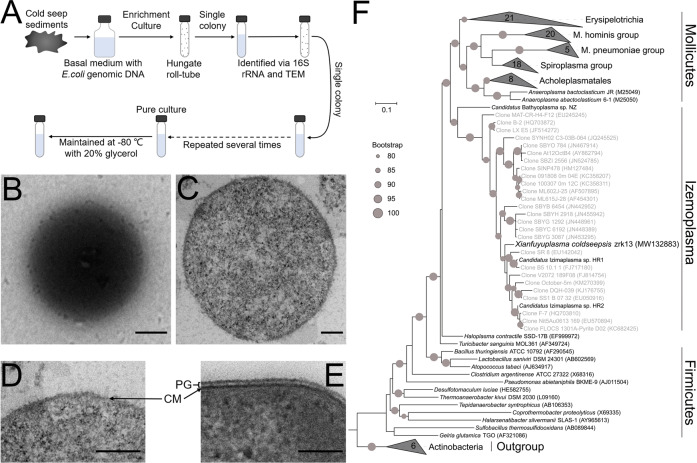
DNA degradation-driven isolation strategy and morphology of *X. coldseepsis* zrk13. **A** Diagrammatic scheme of enrichment and isolation of Izemoplasma bacteria. **B** and **C** TEM observation of strain zrk13. **D** TEM observation of the ultrathin section of strain zrk13. **E** TEM observation of the ultrathin section of a typical Gram-positive bacterium *Clostridium* sp. zrk8. CM cell membrane, PG peptidoglycan; Scale bars, 100 nm. **F** Phylogenetic analysis of *X. coldseepsis* zrk13. Phylogenetic tree of almost complete 16S rRNA gene sequences from Tenericutes and Firmicutes representatives. Some Actinobacteria members were used as the outgroup. The tree is inferred and reconstructed under the maximum likelihood criterion and nodes with >80% bootstrap support are labeled with a gray circle (expressed as percentages of 1000 replications). And the names indicated in gray in quotation represent taxa that are not yet validly published. Bar, 0.1 substitutions per nucleotide position.

Despite what has been learned from cultivation independent methods [5], the lack of cultured free-living representatives of deep-sea *Candidatus* Izemoplasma has hampered a more detailed exploration of the group. With this, we need cultivate representatives to provide further understanding of *Candidatus* Izemoplasma from the deep-sea environment. In service of this goal, we improved the enrichment strategy by using a basal medium supplemented with *E. coli* genomic DNA as the nutrient source, given the previously predicted superior DNA degradation capability of *Candidatus* Izemoplasma [5, 16]. Using this medium, we anaerobically enriched the deep-sea sediment samples at 28 °C for 6 months. Enriched samples were then plated on solid medium in Hungate tubes and individual colonies with distinct morphology were picked and cultured (Fig. 1A). Excitingly, most cultured colonies were identified as members of *Candidatus* Izemoplasma. Among them, strain zrk13 possessed a faster growth rate and was chosen for further study. Under transmission electron microscopy (TEM) observation, the cells of strain zrk13 were coccoid (Fig. 1B, C), ~300–800 nm in size, and had no flagellum. As expected, cells of strain zrk13 had no distinct cell wall, which was confirmed by the TEM observation of ultrathin-sections of strain zrk13 (Fig. 1D) and a typical Firmicutes bacterium (Fig. 1E). Overall, we successfully obtained the first cultured free-living representative of *Candidatus* Izemoplasma from the cold seep through a DNA degradation-driven strategy, which might be useful to enrich and isolate other uncultured candidates of Izemoplasma in the future.

### Genomic characteristics and phylogenetic analysis of strain zrk13

To understand more characteristics of the strain zrk13, its whole genome was sequenced and analyzed. The genome size of strain zrk13 is 1,958,905 bp with a DNA G + C content of 38.2% (Supplementary Fig. 2 and Supplementary Table 3). Annotation of the genome of strain zrk13 revealed it consisted of 1872 predicted genes that included 64 RNA genes (three rRNA genes, 42 tRNA genes and 28 other ncRNAs). The genome relatedness values were calculated by the AAI, the ANI, in silico DNA-DNA similarity (*is*DDH) and the tetranucleotide signatures (Tetra), against four genomes including strains zrk13, HR1, HR2 and ZiA1 (Supplementary Table 3). The AAI values of zrk13 with HR1, HR2 and ZiA1 were 68.8%, 66.5% and 64.3%, respectively. The average nucleotide identities (ANIb) of zrk13 with HR1, HR2 and ZiA1 were 68.72%, 67.42% and 66.57%, respectively. The average nucleotide identities (ANIm) of zrk13 with HR1, HR2 and ZiA1 were 85.94%, 86.53% and 81.98%, respectively. The tetra values of zrk13 with HR1, HR2 and ZiA1 were 0.85192, 0.77694 and 0.837. Based on digital DNA-DNA hybridization employing the GGDC, the in silico DDH estimates for zrk13 with HR1, HR2 and ZiA1 were 17.50%, 18.50% and 15.80%, respectively. These results together demonstrated the genome of strain zrk13 to be clearly below established ‘cut-off’ values (ANIb: 95%, ANIm: 95%, AAI: 95%, *is*DDH: 70%, Tetra: 0.99) for defining bacterial species, strongly suggesting it represents a novel taxon within *Candidatus* Izemoplasma as currently defined.

To clarify the taxonomic status of strain zrk13, we further performed the phylogenetic analyses with 16S rRNA genes from cultured Tenericutes and some typical Firmicutes representatives (Bacilli and Clostridia members), the specific Firmicutes representative (Erysipelotrichia members), unclassified Haloplasma contractile SSD-17B, and some uncultured *Candidatus* Izemoplasma. The maximum likelihood tree of 16S rRNA placed the clade ‘Izemoplasma’ as a sister group of the class Mollicutes, which together form a distinct cluster as Tenericutes separating from Firmicutes except the Erysipelotrichia lineage (Fig. 1F), whose exact taxonomic status is unclear and controversial [2, 47, 48]. Of note, the position of *Candidatus* Izemoplasma was just located between the lineages of typical Firmicutes bacteria and Mollicutes members, indicating *Candidatus* Izemoplasma could represent an intermediate in the reductive evolution from Firmicutes to Mollicutes. Consistently, the genome based tree also placed strain zrk13 and other uncultivated Izemoplasma bacteria together with Mollicutes as a distinct cluster with Firmicutes except the Erysipelotrichia lineage (Supplementary Fig. 3), further confirming the specific taxonomic status of Izemoplasma. Given that strain zrk13 is the first cultured free-living representative of a proposed class *Candidatus* Izemoplasma [5], which is qualified to be named as Izemoplasma from now on. A sequence similarity calculation using the NCBI server indicated that the closest relative of strain zrk13 was uncultivated Izemoplasma HR1 (95.26%). Therefore, we propose that strain zrk13 is classified as the type strain of a novel genus in the class Izemoplasma, for which the name *Xianfuyuplasma coldseepsis* gen. nov., sp. nov. is proposed.

### Description of *Xianfuyuplasma* gen. nov. and *Xianfuyuplasma coldseepsis* sp. nov

For the genus name *Xianfuyuplasma*, Xian.fu.yu’plas.ma. L. fem. n. *Xianfuyu* comes from a strange animal’s name described in the Classic of Mountains and Rivers-a very famous Chinese mythology. *Xianfuyu* is a kind of strange animal possessing a fish’s head and pig’s body. -plasma, formed or molded, refers to the lack of cell wall. The genus *Xianfuyuplasma* is a kind of strictly anaerobic microorganisms, whose cells are non-motile and coccoid. Its phylogenetic position is classified in the family Izemoplasmaceae, order Izemoplasmales, class Izemoplasma within the phylum Tenericutes. The type species is *Xianfuyuplasma coldseepsis*.

*Xianfuyuplasma coldseepsis* (cold.seep’sis. L. gen. pl. n. *coldseepsis* of or belonging to the deep-sea). For this species, cells are coccoid, ~300–800 nm in size, and had no flagellum; strictly anaerobic; the temperature range for growth is 24–32 °C with an optimum at 28 °C (Supplementary Fig. S4A); growing at pH values of 6.0–8.0 (optimum, pH 7.0) (Supplementary Fig. S4B); growth occurs at NaCl concentrations between 0.0 and 8.0% with an optimum growth at 4.0% NaCl (Supplementary Fig. 4C); growth is stimulated by using glucose, maltose, butyrate, acetate, formate, fructose, sucrose, mannose, starch, isomaltose, trehalose, lactate, ethanol, glycerin or rhamnose as a sole carbon source (Supplementary Table 4), showing quite differences toward the carbon sources predicted by other Izemoplasma members. The type strain, zrk13^T^, was isolated from the sediment of deep-sea cold seep, P. R. China.

### Organic nutrient and thiosulfate significantly promote the growth of *X.coldseepsis* zrk13

**Fig. 2:**
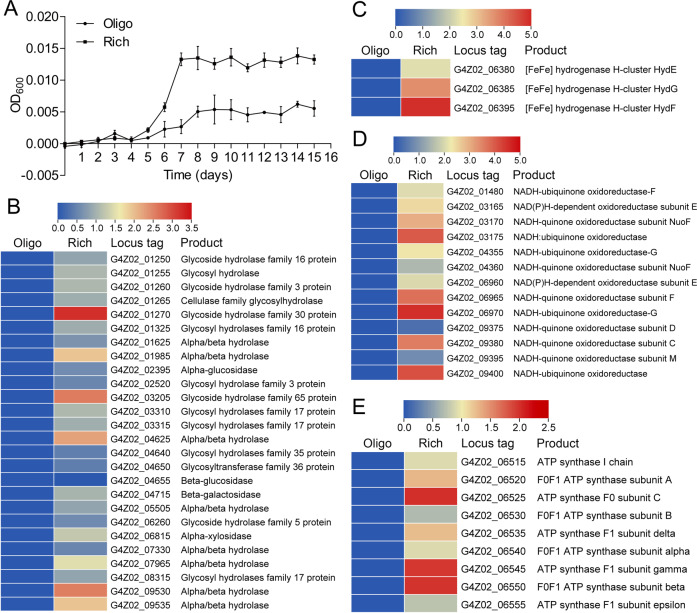
Organic nutrient significantly promotes the growth of *X. coldseepsis* zrk13. **A** Growth assays of strain zrk13 in the oligotrophic and rich media. **B** Transcriptomics based heat map showing all up-regulated genes encoding glycosyl hydrolases. **C** Transcriptomics based heat map showing three up-regulated iron hydrogenase maturation genes (*hydEFG*). **D** Transcriptomics based heat map showing all up-regulated genes encoding NADH-quinone/ubiquinone oxidoreductase. **E** Transcriptomics based heat map showing all up-regulated genes encoding ATP synthase. “Oligo” indicates the oligotrophic medium; “Rich” indicates the rich medium.

In the previous study, uncultivated Izemoplasma HR1 and HR2 were found to be better enriched in the medium amended with small amount of glucose (5 g/L) and yeast extract (0.2 g/L) than that in the basal medium [5]. Similarly, the growth rate of *X. coldseepsis* zrk13 was promoted by about three times in the rich medium containing yeast extract (1.0 g/L) and peptone (1.0 g/L) when compared to that cultured in the oligotrophic medium containing less yeast extract (0.1 g/L) and peptone (0.1 g/L) (Fig. 2A), strongly indicating strain zrk13 is a heterotrophic bacterium. Given that the tricarboxylic cycle was incomplete in the previous [5] and the present Izemoplasma genomes, we sought to ask how does Izemoplasma utilize organic nutrient. Therefore, we performed the transcriptomic analysis of *X. coldseepsis* zrk13 that cultured in oligotrophic and rich media. The results revealed that the expressions of genes encoding different glycoside hydrolases (Fig. 2B), FeFe hydrogenases (Fig. 2C), NADH-ubiquinone oxidoreductase related proteins (Fig. 2D) and ATP synthase (Fig. 2E) were significantly up-regulated. Glycoside hydrolases are enzymes that catalyze the hydrolysis of the glycosidic linkage of glycosides, leading to the formation of small molecules sugars for easy utilization by organisms [49]. Correspondingly, expressions of different glycoside hydrolases including families 1, 16, 17, 30, 35, 65 were evidently up-regulated (Fig. 2B), suggesting many categories of glycans could be efficiently degraded by *X. coldseepsis* zrk13. Indeed, all the present and two uncultured Izemoplasma HR1 and HR2 genomes contained a complete set of genes associated with Embden-Meyerhof-Parnas (EMP) glycolysis and pentose phosphate pathways [5], indicating Izimaplasa bacteria possessed a significant capability of glycan metabolism. Of note, the expressions of all three iron hydrogenase encoding genes (*hydEFG*) were significantly up-regulated (up to 30 folds) (Fig. 2C), and iron hydrogenases were proposed to catalyze the oxidation of hydrogen or the reduction of protons with the transfer of electrons. Moreover, the expressions of many genes related to NADH-ubiquinone oxidoreductase were evidently up-regulated (up to 20 folds) (Fig. 2D), and NADH-ubiquinone oxidoreductases were thought to catalyze the transfer of two electrons from NADH to reduce ubiquinone to ubiquinol and were also the entry point for a large fraction of the electrons that traverse the respiratory chain [50, 51]. With this, it is reasonable to see that the expressions of different genes encoding ATP synthase were also greatly up-regulated (Fig. 2E), and ATP synthases were responsible for converting the energy of protons (H^+^) moving down their concentration gradient into the synthesis of ATP and thereby promoting the bacterial growth. Overall, we conclude that *X. coldseepsis* zrk13 could effectively utilize organic nutrients for growth through anaerobic fermentation and has the potential to produce hydrogen [5].

**Fig. 3:**
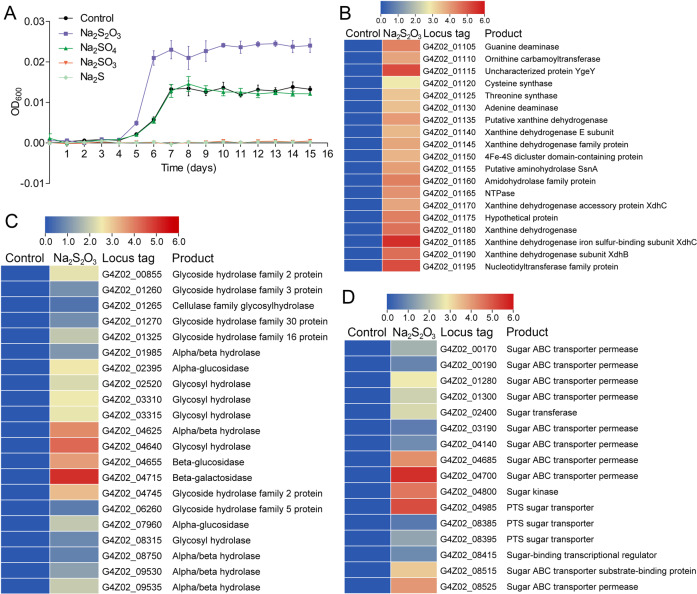
Thiosulfate significantly promotes the growth of *X. coldseepsis* zrk13. **A** Growth assays of strain zrk13 in the modified rich medium supplemented without or with Na_2_SO_4_ (100 mM), Na_2_S_2_O_3_ (100 mM), Na_2_SO_3_ (1 mM), and Na_2_S (1 mM), respectively. **B** Transcriptomics based heat map showing an up-regulated gene cluster encoding Fe-S protein, nucleotide and amino acid metabolisms associated proteins. **C** Transcriptomics based heat map showing all up-regulated genes encoding glycosyl hydrolases. **D** Transcriptomics based heat map showing all up-regulated genes encoding sugar ABC transporter permease.

*X. coldseepsis* zrk13 was isolated from a deep-sea cold seep, and different sulfur sources were detected to ubiquitously exist in the environment in our previous study [22]. Therefore, we tested the effects of different sulfur-containing inorganic substances (e.g., Na_2_SO_4_, Na_2_SO_3_, Na_2_S_2_O_3_, Na_2_S) on the growth of *X. coldseepsis* zrk13. The results showed that the supplement of different concentrations of Na_2_SO_4_ (from 20 to 200 mM) had no evident effects on the growth of strain zrk13. While the supplement of low concentration (1 mM) of Na_2_SO_3_ and Na_2_S was detrimental to the growth of strain zrk13 (Fig. 3A). Only the supplement of Na_2_S_2_O_3_ (100 mM) could significantly promote the growth of strain zrk13 (Fig. 3A). We thus performed the transcriptomic analysis of strain zrk13 that cultured in the modified rich medium amended with or without Na_2_S_2_O_3_ to explore the underlying mechanism of growth promotion. Surprisingly, we didn’t find obvious up-regulation of key genes closely associated with sulfur metabolism that identified in strain zrk13. Alternatively, the expressions of a gene cluster (from G4Z02_01105 to G4Z02_01195; containing genes encoding an Fe-S cluster protein and diverse proteins responsible for nucleotide and amino acid metabolisms) were significantly up-regulated (Fig. 3B). It is known that Fe-S proteins are metal cofactors required for essential biological pathways, including respiration, photosynthesis, elemental cycling, DNA repair and nucleotide metabolisms [52, 53]. Among the proteins associated with nucleotide metabolism, most of them were xanthine dehydrogenase related (Fig. 3B). Xanthine dehydrogenase belongs to the group of molybdenum-containing hydroxylases involved in the oxidative metabolism of purines [54]. In addition, the expressions of genes encoding guanine deaminase, adenine deaminase, NTPase, and nucleotidyltransferase that related to nucleotide metabolism were also evidently up-regulated (Fig. 3B). On the other hand, the expressions of genes encoding ornithine carbamoyltransferase, cysteine synthase, threonine synthase, and aminohydrolase that related to amino acid metabolism were markedly up-regulated (Fig. 3B). Meanwhile, the expressions of large amount of genes encoding enzymes responsible for saccharides degradation (Fig. 3C) and sugar transport (Fig. 3D) were evidently up-regulated. In combination of the results showed in Fig. 2, we speculate that thiosulfate may accelerate the hydrolysis and uptake of saccharides under the control of [Fe-S] associated proteins and thereby synthesizing energy and promoting the growth of strain zrk13. Together, we believe *X. coldseepsis* zrk13 possesses a capability to utilize both organic nutrients (e.g., saccharides, amino acids and nucleic acids) and inorganic sulfur-containing compounds (e.g., thiosulfate) ubiquitously existing in the deep-sea environments.

### *X.coldseepsis* zrk13 possesses a significant capability of degrading DNA in both laboratorial and deep-sea environments

**Fig. 4:**
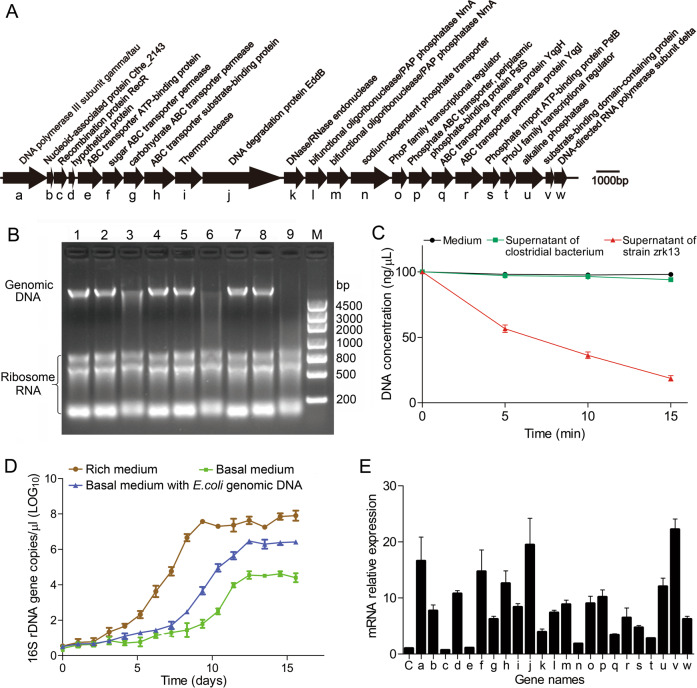
*X. coldseepsis* zrk13 possesses a significant capability of degradation and utilization of extracellular DNA. **A** Gene arrangements of a putative DNA-degradation locus in strain zrk13. The alphabets shown in the *X*-axis indicate the code names of different genes within the gene cluster. **B** Detection of DNA degradation ability of strain zrk13 by agarose gel electrophoresis. Lane 1, lane 4 and lane 7 indicate 2 µg *E. coli* genomic DNA/RNA treated by the medium without cells for 5 min, 10 min and 15 min at 37 °C, respectively. Lane 2, lane 5 and lane 8 indicate 2 µg *E. coli* genomic DNA/RNA treated by the supernatant of a Clostridial bacterium for 5 min, 10 min and 15 min at 37 °C, respectively. Lane 3, lane 6 and lane 9 indicate 2 µg *E. coli* genomic DNA/RNA treated by the supernatant of strain zrk13 for 5 min, 10 min and 15 min at 37 °C, respectively. M, DL4500 molecular weight DNA marker. **C** Quantification of DNA degradation by strain zrk13. DNA concentrations after degradation by the medium and zrk13 supernatant as shown in (**B**) were determined by Nanodrop. Three replicates were performed. **D** Growth assays of strain zrk13 cultivated in the rich medium and basal medium supplemented with or without 1 µg/mL *E. coli* genomic DNA/RNA. **E** qRT-PCR detection of expression changes of genes shown in (**A**) when strain zrk13 was cultivated in the basal medium supplemented with or without 1 µg/mL *E. coli* genomic DNA/RNA. Three biological replicates were performed. The code names shown in the *X*-axis indicate the gene names shown in (**A**). “C” indicates control.

Based on several previous reports [2, 5, 16] and our present isolation strategy, Izemoplasma is believed to degrade extracellular DNA and use as a nutrient source for growth. However, this hypothesis is still lack of solid proof due to the uncultivation status of Izemoplasma. With that, detection of DNA degradation and utilization was further performed with strain zrk13. First, an in-depth analysis of the genome of strain zrk13 revealed the existence of a putative DNA-degradation related gene cluster, which contained genes encoding DNA degradation protein EddB, DNase/RNase endonuclease, thermonuclease, phosphohydrolases, ABC-transporters, phosphate transporters and other proteins associated with DNA degradation (Fig. 4A). In order to test the ability of strain zrk13 to degrade DNA, *E. coli* genomic DNA without removing RNA was extracted for further assays. The results showed that the supernatant of zrk13 cultures possessed an obvious degradation effect toward *E. coli* genomic DNA within 5 min (Fig. 4B, lane 3), leading to the DNA concentration decreasing from 100 to 56.5 ng/µL (Fig. 4C); and degraded almost all DNA within 15 min (Fig. 4B, lane 9), leading to the DNA concentration decreasing from 100 to 18.6 ng/µL (Fig. 4C) at 37 °C. However, the supernatant of zrk13 didn’t degrade RNA at all, indicating its specific DNA degradation capability (Fig. 4B). In comparison, the supernatant of zrk13 showed a relative slower degradation rate at 4 °C, leading to the DNA concentration decreasing from 100 to 39 and 16 ng/µL after 6 h and 12 h treatment, respectively (Supplementary Fig. S5). Moreover, the supplement of genomic DNA in the basal medium could significantly promote the growth rate and biomass amount of zrk13 (Fig. 4D), strongly suggesting this bacterium could efficiently utilize the digested DNA as a nutrient source to support further growth. Consistently, the expressions of genes related to DNA degradation within the gene cluster shown in Fig. 4A were significantly up-regulated, especially the gene encoding DNA degradation protein EddB, which was up-regulated about 20-fold (Fig. 4E). Notably, compared with other 62 genomes of Tenericutes and Firmicutes bacteria, the members of Izemoplasma harbored more genes associated with DNA degradation than those in Mollicutes and Firmicutes (Supplementary Table 5 and Supplementary Fig. 6), confirming the strong DNA degradation capability of Izemoplasma bacteria.

**Fig. 5:**
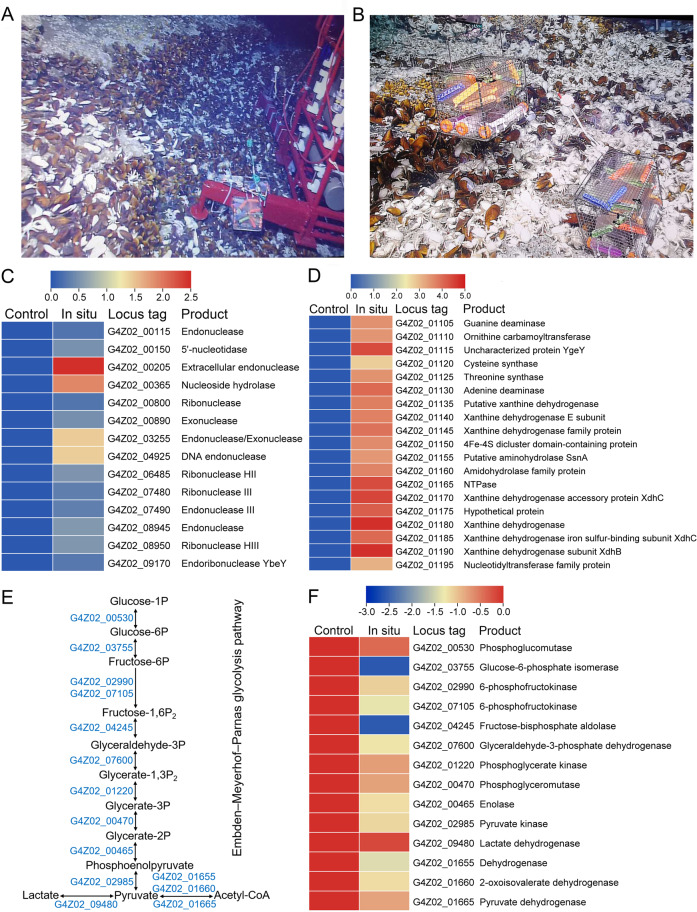
Transcriptomics analysis of *X. coldseepsis* zrk13 incubated in the deep-sea cold seep. Distant (**A**) and close (**B**) views of the in situ experimental apparatus in the deep-sea cold seep where distributed many mussels and shrimps. **C** Transcriptomics based heat map showing all up-regulated genes encoding enzymes degrading nucleic acids after a 10-day incubation of strain zrk13 in the deep-sea cold seep. **D** Transcriptomics based heat map showing an up-regulated gene cluster encoding Fe-S protein, nucleotide and amino acid metabolisms associated proteins. **E** Diagrammatic scheme of EMP glycolysis pathway. The gene numbers showing in this scheme are the same with those shown in (**F**). **F** Transcriptomics based heat map showing all down-regulated genes associated with EMP glycolysis pathway after a 10-day incubation of strain zrk13 in the deep-sea cold seep.

Taken all the above results, we are clear about the metabolic landscape of *X. coldseepsis* zrk13 based on the laboratorial conditions. However, its actual metabolisms performed in the deep-sea environment are still obscure. With that, we performed the in situ cultivation in the June of 2020 to study the metabolisms of strain zrk13 in the deep-sea cold seep, where we isolate this bacterium and lives a lot of typical cold seep animals such as mussels and shrimps (Fig. 5A, B). The transcriptomic results based on the in situ cultivated cells showed that the expressions of many genes encoding exonuclease, endonuclease and ribonuclease were obviously up-regulated compared to the control group (Fig. 5C), indicating DNA-degradation indeed happened in the deep sea. Notably, the expressions of genes within the Fe-S protein containing gene cluster (as shown in Fig. 3B) were all significantly up-regulated (Fig. 5D). Given that this gene cluster is closely associated with nucleotide and amino acid metabolisms, we firmly believe strain zrk13 possesses a remarkable capability to degrade nucleotide and amino acid and then obtain energy to maintain its growth in situ. In comparison, the expressions of almost all the genes involved in EMP glycolysis were significantly down-regulated (Fig. 5E, F), which might be due to the deficiency of organic nutrients in the deep-sea environment. Instead, strain zrk13 could be able to maintain a good growth status mainly by digesting DNA to obtain energy for supporting growth given its strong DNA degradation capability and the ubiquitous existence of extracellular DNA in the deep-sea sediments [16, 20].

## Discussion

Microbes in deep ocean sediments represent a large portion of the biosphere, and resolving their ecology is crucial for understanding global ocean processes [55–57]. Despite the global importance of these microorganisms, majority of deep-sea microbial diversity remains uncultured and poorly characterized [55]. Description of the metabolisms of these novel taxa is advancing our understanding of their biogeochemical roles, including the coupling of elements and nutrient cycling, in the deep oceans. Given the importance of these communities to the oceans, there is an urgent need to obtain more uncultivated isolates for better resolving the diversity and ecological roles of these uncultured taxa. *Candidatus* Izemoplasma bacteria were proposed to represent a novel class of free-living representatives from a Tenericutes clade found in deep-sea methane seeps [5], and they were believed to actively participate in the primary degradation of extracellular DNA in anoxic marine sediments and contribute to the biogeochemical cycling of deep biosphere [5, 16]. Unfortunately, up to date, there is no any available pure culture of Izemoplasma bacteria, which seriously hinders the accurate determination of their features such as growth, metabolism, physiology, and ecology.

**Fig. 6:**
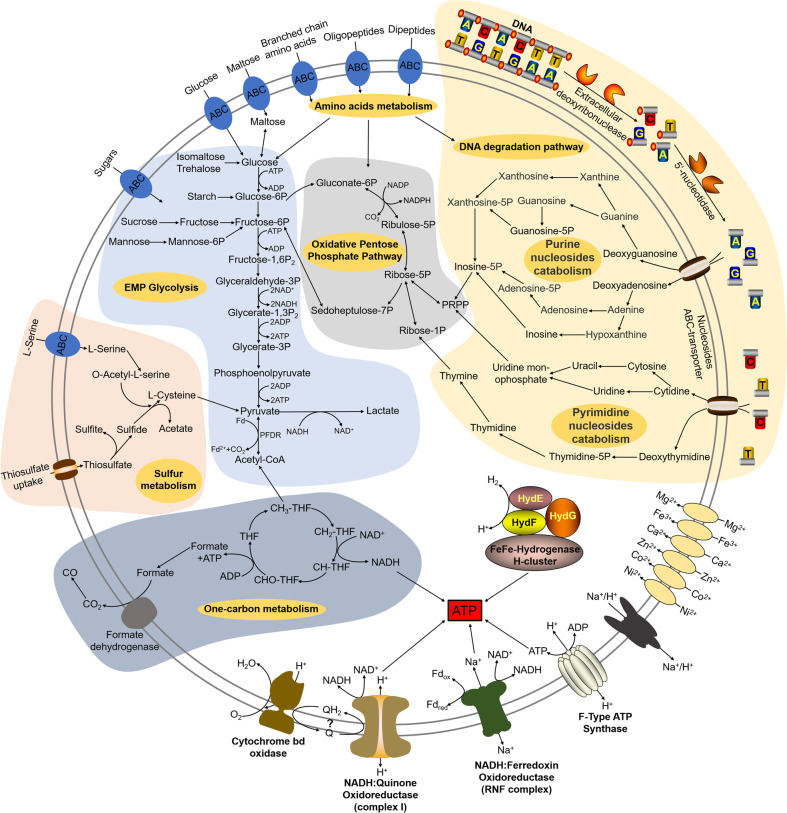
Muti-omics based central metabolisms model of *X. coldseepsis* zrk13. In this model, central metabolisms including DNA degradation, EMP glycolysis, oxidative pentose phosphate pathway, hydrogen production, electron transport system, sulfur metabolism and one carbon metabolism are shown. All the above items are closely related to the energy production in *X. coldseepsis* zrk13. In detail, strain zrk13 contains a complete set of genes related to DNA-degradation, which could catabolically exploit various sub-components of DNA, especially purine-based molecules. And further degradation into nucleotides and nucleosides by nucleases were required to facilitate the introduction of DNA sub-components into cells. Once imported into the cytoplasm, purine- and pyrimidine-deoxyribonucleosides were further broken down into different bases, whereby the respective bases may enter catabolic pathways. The metabolites of nucleosides catabolism were finally transformed into phosphoribosyl pyrophosphate (PRPP) and Ribose-1P, thus entering the oxidative pentose phosphate pathway, which is closely related to EMP glycolysis pathway. Sulfide generated by sulfur reduction from thiosulfate works together with the L-serine to form acetate and L-cysteine, which eventually enter the pyruvate synthesis pathway. Furthermore, the formate produced in one carbon metabolism was converted into CO_2_/CO by formate dehydrogenase. Iron hydrogenases catalyze the reduction of protons to hydrogen for the energy production. A membrane-bound, Na^+^-transporting NADH: Ferredoxin oxidoreductase (RNF complex), the H^+^-transporting NADH: Quinone oxidoreductase (complex I) and F-type ATP synthase required for energy metabolism are present in strain zrk13 genome. Both complex I and the cytochrome bd oxidase interact with the quinone pool, which are associated with energy production.

In the present study, we developed an efficient enrichment method driven by DNA-degradation (Fig. 1A), and successfully obtained a novel Izemoplasma isolate, *X. coldseepsis* zrk13, which is the first pure culture in the Izemoplasma class. Based on the isolate, we detailedly explored its physiology, genomic traits, phylogenetics and metabolisms through bioinformatics, biochemical and transcriptomic methods, and proposed a model describing its central metabolic pathways (Fig. 6). Clearly, DNA and organic matter degradation and utilization play key roles for energy production and thereby promoting the growth of *X. coldseepsis* zrk13 (Fig. 6).

In the laboratorial culturing condition, *X. coldseepsis* zrk13 prefers performing a heterotrophic life given the easily available organic matter present in the medium (Fig. 2A). Therefore, the expressions of large amount of genes associated with sugar degradation were significantly up-regulated in strain zrk13 that cultured in the rich medium when compared to that cultured in the oligotrophic medium (Fig. 2B). In contrast, the expressions of genes responsible for sugar metabolisms (EMP glycolysis) were evidently down-regulated in strain zrk13 that cultivated in situ (Fig. 5E, F) when compared to that incubated in the rich medium. Considering the exchanges between the medium inside the incubation bag and the outside seawater, we speculate that the down-regulation of saccharide metabolism in situ might be the lower content of organic matters in the seawater than that in the rich medium. Notably, thiosulfate also promotes the saccharides metabolism and thereby benefiting the bacterial growth (Fig. 3), which endows *X. coldseepsis* zrk13 a better adaptability to the deep-sea environment given ubiquitous existence of thiosulfate in the cold seep [22]. Therefore, we believe saccharide metabolism is crucial for maintaining the growth of *X. coldseepsis* zrk13 both in the laboratorial and deep-sea environments. In combination of previous report [5], we predict part of the ecological role of Izemoplasma in the cold seep is likely in the fermentation of products from the degradation of organic matter to produce lactate and possibly other small molecules, which in turn are utilized by other members of the cold seep microbes (Fig. 6).

In comparison, the degradation of DNA mediated by *X. coldseepsis* zrk13 happened in both laboratorial (Fig. 4) and deep-sea conditions (Fig. 5), strongly indicating this bacterium is indeed a potential DNA degrader. Consistently, an intact locus containing genes encoding enzymes for further digestion of DNA into oligonucleotides and nucleosides, as well as removal of phosphates from nucleotides is identified in the genome of *X. coldseepsis* zrk13 (Fig. 4A). And nucleases within and outside of this gene cluster were demonstrated to function in the course of DNA degradation (Figs. 4 and 5). Notably, the expressions of genes within an Fe-S protein containing gene cluster associated with nucleotide metabolisms were significantly activated in both conditions of thiosulfate supplement (Fig. 3B) and deep sea (Fig. 5D). Given thiosulfate is a ubiquitously distributed sulfur source in the deep-sea cold seep [22], we speculate thiosulfate might be an essential factor promoting strain zrk13 to digest and utilize DNA. Comparative genomics indicated that DNA can be digested by diverse members of Izemoplasma [16] as well as *X. coldseepsis* zrk13 (Supplementary Fig. 6), strongly suggesting Izemoplasma bacteria are specialized DNA-degraders that encode multiple extracellular nucleases. DNA degradation process is closely associated with amino acids and saccharides metabolisms (Fig. 6) as well as the production of acetyl-CoA [5]. The products of nucleic acids (such as urea and ammonium, CO_2_ and acetate) are also important nutrients for other members of microbial communities [5, 16].

It is noting that extracellular DNA is a major macromolecule in global element cycles, and is a particularly crucial source of phosphorus, nitrogen and carbon for microorganisms in the seafloor [16]. Therefore, extracellular available DNAs and their corresponding degraders like Izemoplasma contribute to oceanic and sedimentary biogeochemical cycles to some extent, and provide a potential energy source for microbial communities. Overall, based on the comprehensive investigations of the first cultured isolate of Izemoplasma in both laboratorial and deep-sea conditions, our present study expands the ecophysiological understanding of Izemoplasma bacteria by showing that they actively participate in the primary degradation of extracellular DNA and other organic matter in the deep-sea sediments. Given that strain zrk13 is a strict anaerobic bacterium, future efforts will be required to test how does it degrade extracellular DNA and other organic matter in the deep-sea environment where existing low concentrations of oxygen.

## Supplementary information

Supplementary information

## Data Availability

The whole 16S rRNA sequence of *X*. *coldseepsis* zrk13 has been deposited in the GenBank database (accession number MW132883). The complete genome sequence of *X*. *coldseepsis* zrk13 has been deposited at GenBank under the accession number CP048914. The raw sequencing reads for transcriptomic analysis have been deposited to NCBI Short Read Archive (accession numbers: PRJNA664657, PRJNA669477 and PRJNA669478). The raw amplicon sequencing data have also been deposited to NCBI Short Read Archive (accession number: PRJNA675395).
