# Supplementary material for: Characterization of the first cultured free-living representative of Candidatus Izemoplasma uncovers its unique biology
Source: ISME J. 2021 Mar 21;15(9):2676–91. doi: 10.1038/s41396-021-00961-7 (PMC8397711; doi:10.1038/s41396-021-00961-7)
Supplement: Supplementary file 1 — Supplementary information [file 41396_2021_961_MOESM1_ESM.pdf]

Supplementary information

Characterization of the first cultured free-living representative of *Candidatus* Izemoplasma uncovers its unique biology

Rikuan Zheng[1,2,3,4], Rui Liu[1,2,4], Yeqi Shan[1,2,3,4], Ruining Cai[1,2,3,4], Ge Liu[1,2,4], Chaomin Sun[1,2,4*]

[1]CAS and Shandong Province Key Laboratory of Experimental Marine Biology & Center of Deep Sea Research, Institute of Oceanology, Chinese Academy of Sciences, Qingdao, 266071, China

[2]Laboratory for Marine Biology and Biotechnology, Qingdao National Laboratory for Marine Science and Technology, Qingdao, 266071, China

[3]College of Earth Science, University of Chinese Academy of Sciences, Beijing, 100049, China

[4]Center of Ocean Mega-Science, Chinese Academy of Sciences, Qingdao, 266071, China

[*] Corresponding author

Chaomin Sun Tel.: +86 532 82898857; fax: +86 532 82898857.

E-mail address: sunchaomin@qdio.ac.cn

Supplementary methods

Transcriptional profiling of *X. coldseepsis* zrk13 cultured in different conditions

(1) Library preparation for strand-specific transcriptome sequencing

A total amount of 3 μg RNA per sample was used as input material for the RNA sample preparations. Sequencing libraries were generated using NEBNext® Ultra™ Directional RNA Library Prep Kit for Illumina® (NEB, USA) following manufacturer's recommendations and index codes were added to attribute sequences to each sample. rRNA is removed using a specialized kit that leaves the mRNA. Fragmentation was carried out using divalent cations under elevated temperature in NEBNext First Strand Synthesis Reaction Buffer (5×). First strand cDNA was synthesized using random hexamer primer and M-MuLV Reverse Transcriptase (RNaseH$^-$). Second strand cDNA synthesis was subsequently performed using DNA Polymerase I and RNase H. In the reaction buffer, dNTPs with dTTP were replaced by dUTP. Remaining overhangs were converted into blunt ends via exonuclease/polymerase activities. After adenylation of 3' ends of DNA fragments, NEBNext Adaptor with hairpin loop structure was ligated to prepare for hybridization. In order to select cDNA fragments of preferentially 150~200 bp in length, the library fragments were purified with AMPure XP system (Beckman Coulter, Beverly, USA). Then 3 μL USER Enzyme (NEB，USA) was used with size-selected, adaptor-ligated cDNA at 37 ℃ for 15 min followed by 5 min at 95 ℃ before PCR. Then PCR was performed with Phusion High-Fidelity DNA polymerase, Universal PCR primers and

Index (X) Primer. At last, products were purified (AMPure XP system) and library quality was assessed on the Agilent Bioanalyzer 2100 system.

(2) Clustering and sequencing

The clustering of the index-coded samples was performed on a cBot Cluster Generation System using TruSeq PE Cluster Kit v3-cBot-HS (Illumia) according to the manufacturer's instructions. After cluster generation, the library preparations were sequenced on an Illumina Hiseq platform and paired-end reads were generated.

(3) Data analysis

Raw data (raw reads) of fastq format were firstly processed through in-house perl scripts. In this step, clean data (clean reads) were obtained by removing reads containing adapter, reads containing ploy-N and low quality reads from raw data. At the same time, Q20, Q30 and GC content the clean data were calculated. All the downstream analyses were based on the clean data with high quality. Reference genome and gene model annotation files were downloaded from genome website directly. Both building index of reference genome and aligning clean reads to reference genome were used Bowtie2-2.2.3 [1]. HTSeq v0.6.1 was used to count the reads numbers mapped to each gene. And then FPKM of each gene was calculated based on the length of the gene and reads count mapped to this gene. FPKM, expected number of Fragments Per Kilobase of transcript sequence per Millions base pairs sequenced, considers the effect of sequencing depth and gene length for the reads

count at the same time, and is currently the most commonly used method for estimating gene expression levels [2].

(4) Differential expression analysis

Differential expression analysis of two conditions/groups (two biological replicates per condition) was performed using the DESeq R package (1.18.0) [3]. DESeq provide statistical routines for determining differential expression in digital gene expression data using a model based on the negative binomial distribution. The resulting *P*-values were adjusted using the Benjamini and Hochberg's approach for controlling the false discovery rate. Genes with an adjusted *P*-value < 0.05 found by DESeq were assigned as differentially expressed. (For DEGSeq without biological replicates) Prior to differential gene expression analysis, for each sequenced library, the read counts were adjusted by edgeR program package through one scaling normalized factor. Differential expression analysis of two conditions was performed using the DEGSeq R package (1.20.0) [4]. The *P* values were adjusted using the Benjamini & Hochberg method. Corrected *P*-value of 0.005 and $\log_2$ (Fold change) of 1 were set as the threshold for significantly differential expression.

(5) GO and KEGG enrichment analysis of differentially expressed genes

Gene Ontology (GO) enrichment analysis of differentially expressed genes was implemented by the GOseq R package, in which gene length bias was corrected [5]. GO terms with corrected *P* value less than 0.05 were considered significantly enriched by differential expressed genes. KEGG is a database resource for

understanding high-level functions and utilities of the biological system, such as the cell, the organism and the ecosystem, from molecular-level information, especially large-scale molecular datasets generated by genome sequencing and other high-throughput experimental technologies (http://www.genome.jp/kegg/) [6]. We used KOBAS software to test the statistical enrichment of differential expression genes in KEGG pathways.

References

1. Langmead B, Salzberg SL. Fast gapped-read alignment with Bowtie 2. Nat Methods. (2012); 9: 357-U354.
2. Trapnell C, Pachter L, Salzberg SL. TopHat: discovering splice junctions with RNA-Seq. Bioinformatics. (2009); 25: 1105-1111.
3. Anders S, Huber W. Differential expression analysis for sequence count data. Genome Biol. (2010); 11.
4. Wang LK, Feng ZX, Wang X, Wang XW, Zhang XG. DEGseq: an R package for identifying differentially expressed genes from RNA-seq data. Bioinformatics. (2010); 26: 136-138.
5. Young MD, Wakefield MJ, Smyth GK, Oshlack A. Gene ontology analysis for RNA-seq: accounting for selection bias. Genome Biol. (2010); 11.
6. Kanehisa M, Araki M, Goto S, Hattori M, Hirakawa M, Itoh M, et al. KEGG for linking genomes to life and the environment. Nucleic Acids Res. (2008); 36: D480-D484.

Supplementary results

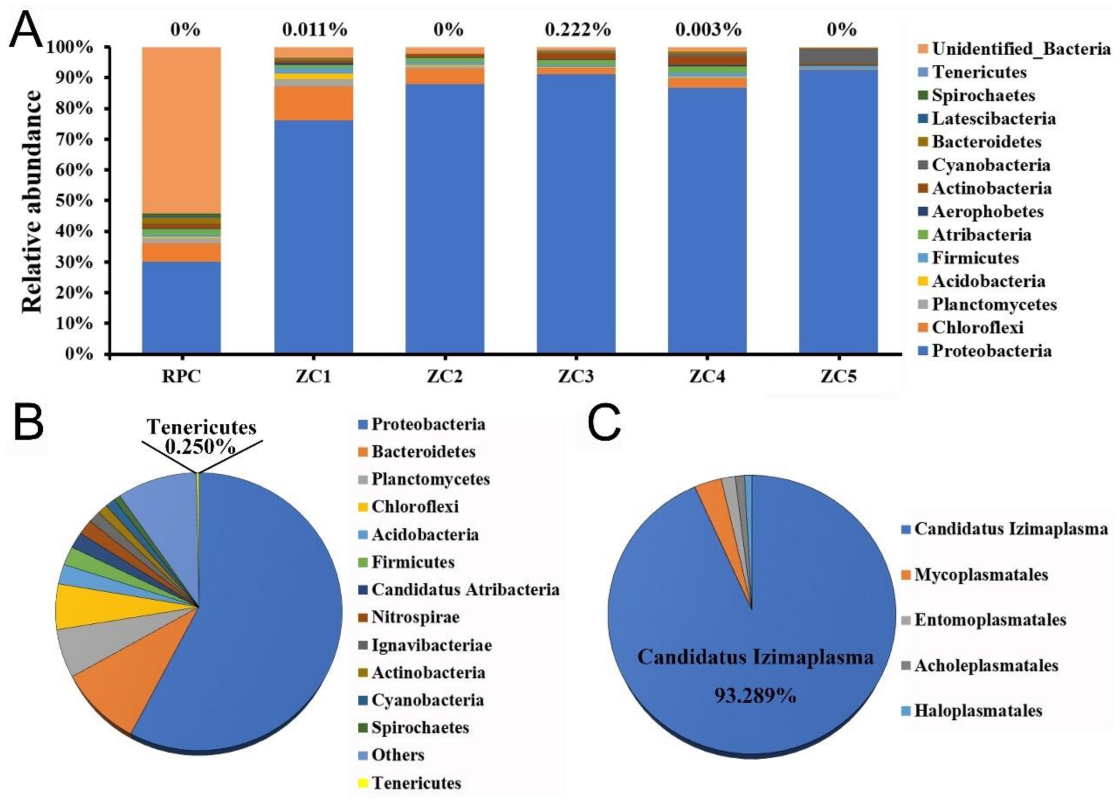

Supplementary Fig 1. Quantification of the abundance of Tenericutes and *Candidatus* Izemoplasma in the deep-sea cold seep. (A) The community structure of six sampling sites in the cold seep sediments as revealed by 16S rRNA gene amplicon profiling. The relative abundances of operational taxonomic units (OTUs) representing different bacteria are shown at the phylum level. (B) Quantification of the abundance of Tenericutes in the bacteria domain based on the metagenomics sequencing. (C) Quantification of the abundance of *Candidatus* Izemoplasma in the phylum Tenericutes based on the metagenomics sequencing.

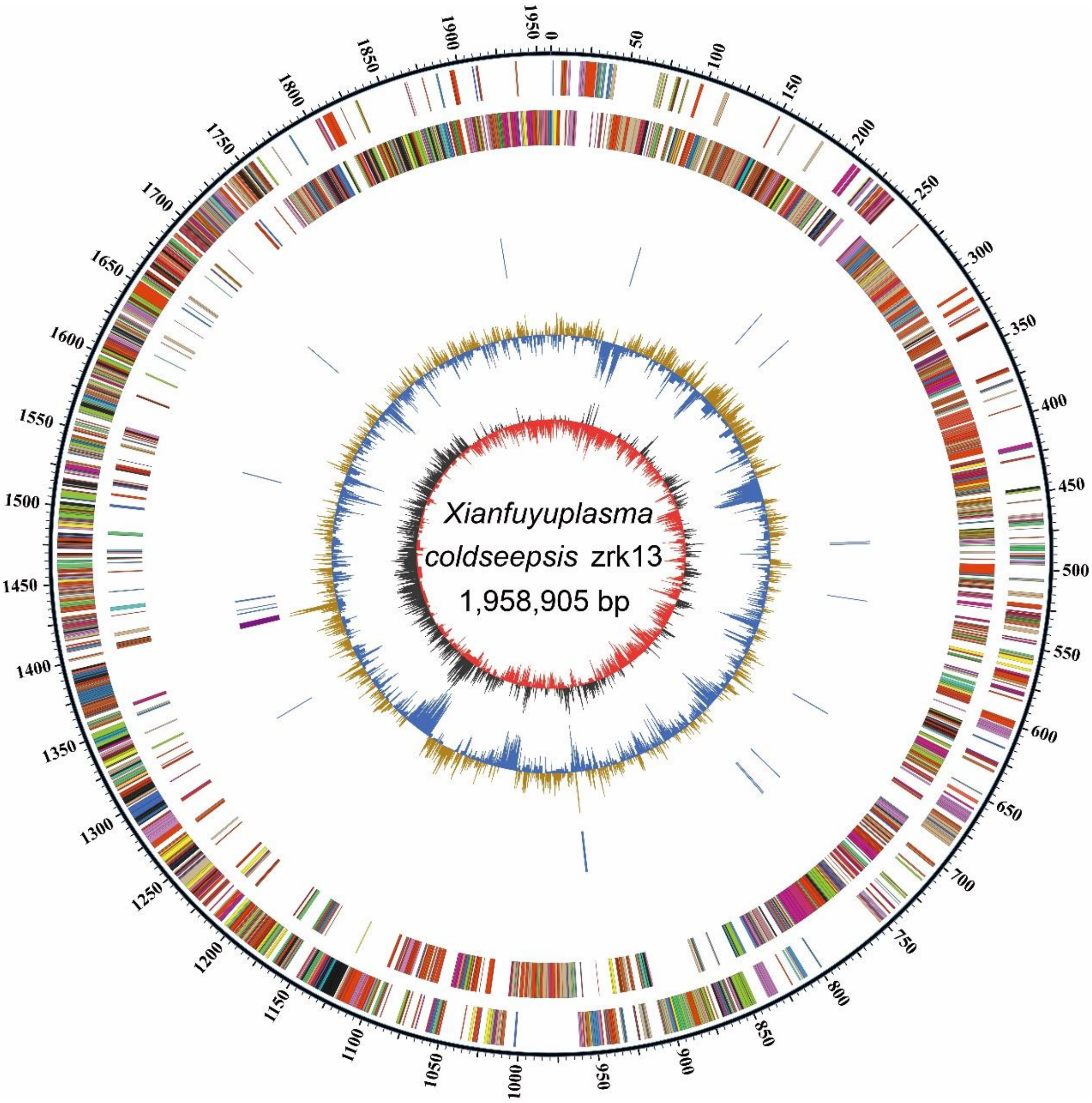

Supplementary Fig 2. Circular diagram of the genome of *X.coldseepsis* zrk13. Rings indicate, from outside to the center: a genome-wide marker with a scale of 50 kb; forward strand genes, colored by COG category; reverse strand genes, colored by COG category; repetitive sequences; RNA genes (tRNAs blue, rRNAs purple); GC content; GC skew.

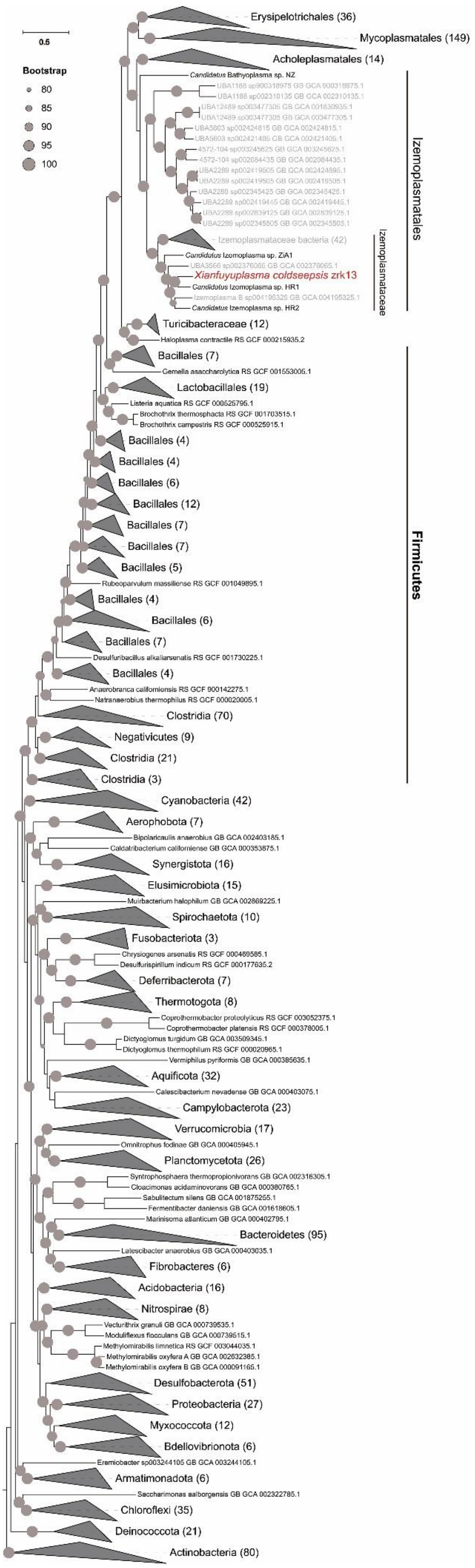

Erysipelotrichales (36)
Mycoplasmatales (149)
Acholeplasmatales (14)
0.5
Bootstrap
80
85
90
95
100
Izemoplasmatales
Izemoplasmataceae
Izemoplasmataceae bacteria (42)
Xianfuyuplasma coldseepsis zrk13
Turicibacteraceae (12)
Bacillales (7)
Lactobacillales (19)
Bacillales (4)
Bacillales (4)
Bacillales (6)
Bacillales (12)
Bacillales (7)
Bacillales (7)
Bacillales (5)
Firmicutes
Bacillales (4)
Bacillales (6)
Bacillales (7)
Bacillales (4)
Clostridia (70)
Negativicutes (9)
Clostridia (21)
Clostridia (3)
Cyanobacteria (42)
Aerophobota (7)
Synergistota (16)
Elusimicrobiota (15)
Spirochaetota (10)
Fusobacteriota (3)
Deferribacterota (7)
Thermotogota (8)
Aquificota (32)
Campylobacterota (23)
Verrucomicrobia (17)
Planctomycetota (26)
Bacteroidetes (95)
Fibrobacteres (6)
Acidobacteria (16)
Nitrospirae (8)
Desulfobacterota (51)
Proteobacteria (27)
Myxococcota (12)
Bdellovibrionota (6)
Armatimonadota (6)
Chloroflexi (35)
Deinococcota (21)
Actinobacteria (80)

Supplementary Fig 3. Maximum likelihood phylogenetic tree of strain zrk13 was reconstructed using the Genome Taxonomy Database Toolkit (https://github.com/Ecogenomics/GTDBTk) based on the concatenated alignment of 120 ubiquitous single-copy proteins. Some Actinobacteria members were used as the outgroup. The tree is inferred and reconstructed under the maximum likelihood criterion and nodes with greater than 80% bootstrap support are labeled with a grey circle (expressed as percentages of 1,000 replications). Bar, 0.5 substitutions per nucleotide position.

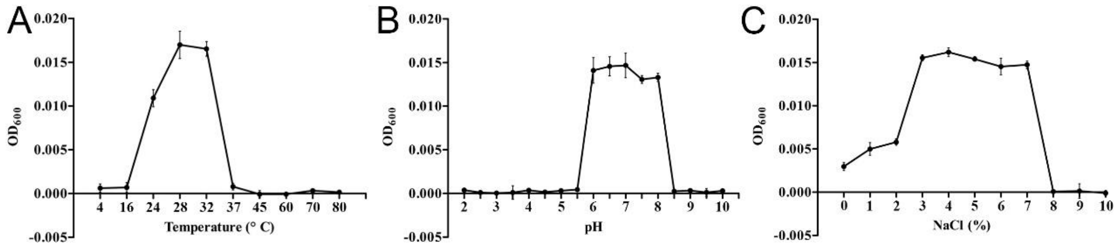

Supplementary Fig 4. The growth curves of strain zrk13 at different temperatures (A), pH (B) and NaCl concentrations (C).

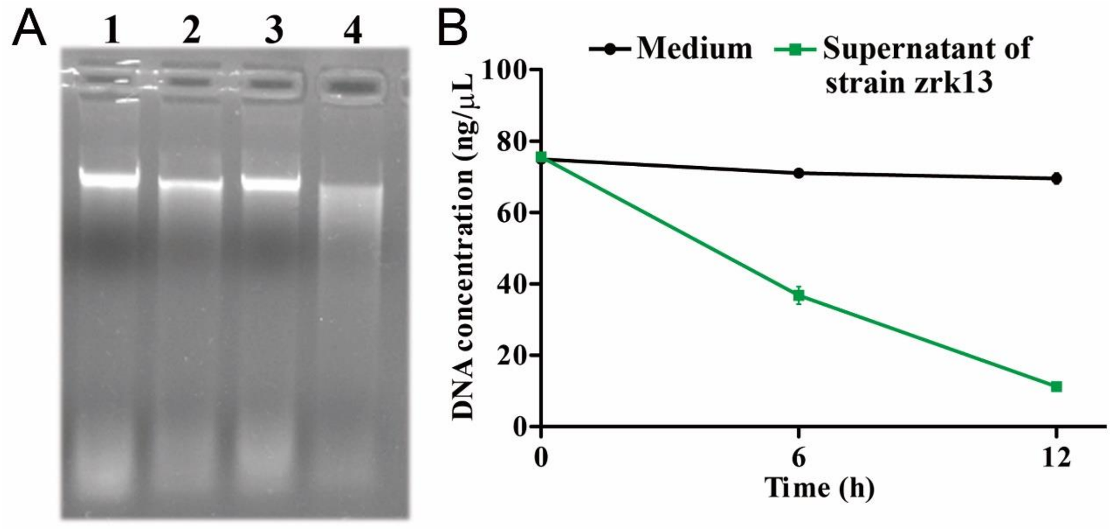

Supplementary Fig 5. Detection of the DNA degradation capability of strain zrk13 at 4 ℃. (A) Detection of the ability of DNA degradation by agrose gel electrophoresis. Lane 1 and lane 3 indicate 1.5 μg *E. coli* genomic DNA treated by the medium without cells (as control) for 6 h and 12 h at 4 ℃, respectively. Lane 2 and lane 4 indicate 1.5 μg *E. coli* genomic DNA treated by zrk13 supernatant for 6 h and 12 h at 4 ℃, respectively. (B) Quantification of DNA degradation by strain zrk13. The concentrations of DNA after degradation by medium and zrk13 supernatant as shown in panel A were determined by Nanodrop. Three replicates were performed.

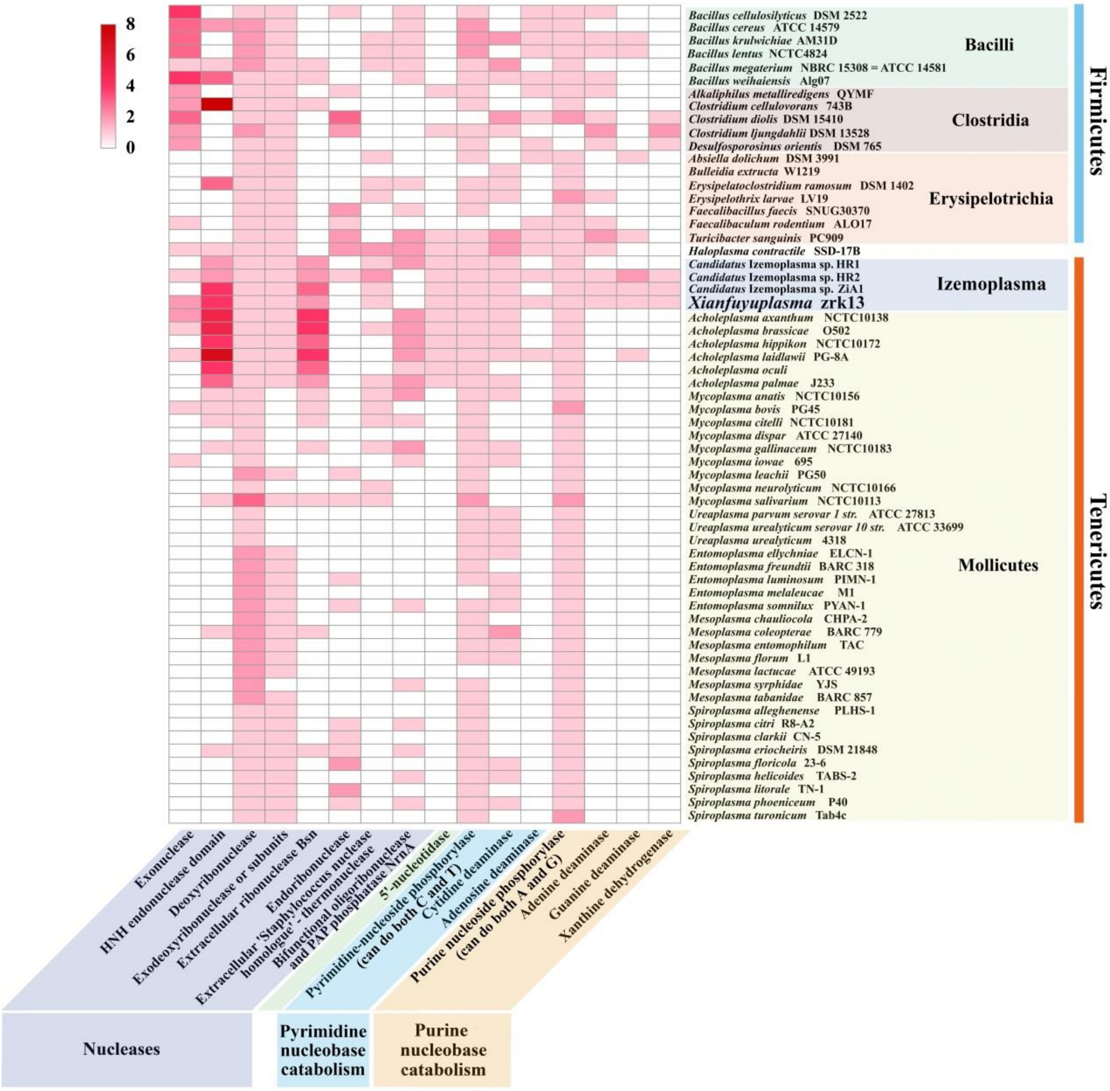

Supplementary Fig 6. Distribution of key genes associated with DNA degradation in the genomes of typical species of phylums Tenericutes and Firmicutes.

Supplementary Table 1. Chemical parameters of the deep-sea cold seep.

Chemical parameters for sampling site	Cold seep
Depth under the surface of ocean (m)	1139
Depth under the surface of sediment (cm)	0~250
Longitude and latitude	E 119 °17'09.106"<br>N 22 °06'55.114"
pH	7.62
Temperature (℃)	3.64
Salinity (‰)	34.49
Dissolved oxygen (mg/L)	3.03
CO_2 (PPM)	828
CH_4 (μM)	2642
Sulfate (mM)	28
S (PPM)	3452.3

Supplementary Table 2. Primers used for qRT-PCR.

Primer name	Nucleotide Sequence (5'-3')
16S-f	CCTGCCCCAAAGTTGAGGAT
16S-r	AGCGCAAGACCATCCCATAG
803-f	TCGGATGATGCGACCTGATG
803-r	CCAAACATCGGTTGGCAAGG
804-f	TCACGATTGATCCCGATGCT
804-r	CATTACGCTTTCGGTTTCGGT
805-f	CCGTCGCTTATTAGAGGGCA
805-r	TAGAGTTTTCGTCGCCCCTC
806-f	TGATGGATTGGATGCAGTAACACT
806-r	AGAGTCCAACAAGGATACCTACAAC
807-f	GGGACCCTTACCGTTGATGT
807-r	GTGAGCCGAATCCACGATTG
808-f	TGTTTGGGCCAAGTATGGGT
808-r	GTACCCGCACGACCGATTT
809-f	ACGCGTATACCGAACTTGCT
809-r	CTTCGTGATACCCCACGCAT
810-f	ATGACAGTCGGTTCTACCGC
810-r	TTTGGTCCTTGTTGCATCGC
811-f	CATACACCGGGGCAAACAAC
811-r	TCTTCACCCCAGATTCGACG
812-f	TTACGGCAACCATCACCCTC
812-r	CTCAACAACGTCACCAACGG
813-f	TGTGAAACAAGGCTTGGCAC
813-r	CGCGTAATCTTCAGCGATGTG
814-f	ATCATCTTGCGGCTACACGG
814-r	GTTCATGTCTGCGCCTTGAC

815-f	ACATGGCCGAACAAAGAGGT
815-r	CGATAGCCAAAGCCCCTTCA
816-f	CAACGGGCGCAATATTCTGG
816-r	TCCGGGCACGAGTTTTGTTA
817-f	TGGAGACTCTCGTGTTGTCG
817-r	AGTTTGTATCCTACACCGCGT
818-f	TATCCGAAGCATTCGCACCA
818-r	TGCATTGTCGCCATCCTTTTC
819-f	TTCGTAGTGTGCGAAGCGAT
819-r	ATTCCGGATTTCGCACTGGT
820-f	ATTTCCGTAGCGCATCCCTC
820-r	CCGATTGCGAGAAGTGTTGC
821-f	AACCCACCAGTGCCCTAGAT
821-r	TCGAGCAGCTTGTTGCATTG
822-f	ATGGAGGCATTTGAGCGTGA
822-r	TCGTCAGAAGCGGTTTTTGC
823-f	TCGTGATGGAATCTCAGCGG
823-r	TCGTAATCGACGCGATCCAA
824-f	ACAGCTACGTACCAGGTTTACG
824-r	TGGGTGGTATTGACAAGGTTCT
825-f	GAAGAACCCGAAGTGGTCGT
825-r	CAGCTGGTTCGATAGCTGGT

Supplementary Table 3. Genomic features of *X. coldseepsis* zrk13 with other uncultivated Izemoplasma strains HR1, HR2 and ZiA1.

Feature	zrk13	HR1	HR2	ZiA1
Gene Bank ID	CP048914	CP009415	JRFF00000000	NQYJ00000000
Genome size (bp)	1,958,905	1,878,735	2,115,618	1,884,011
G+C content (%)	38.2	31.3	29.2	29.6
No.scaffolds/contigs	1	1	78	22
No. of genes	1,872	1,846	2,284	1,877
No. of rRNAs	3	4	3	0
No. of tRNAs	42	38	58	34
No. of protein-coding genes	1,762	1,794	2,222	1,803
Completeness (%)	100	100	92.1	98.7
AAI (%)	100	68.8	66.5	64.3
ANIb (%)	100	68.72	67.42	66.57
ANIm (%)	100	85.94	86.53	81.98
Tetra	1	0.85192	0.77694	0.837
*is*DDH (%)	100	17.50	18.50	15.80

Supplementary Table 4. Physiological characteristics of *X. coldseepsis* zrk13.

**Characteristic**	**zrk13**
Cell length (μm)	0.3-0.8
Temperature range for growth (℃)	24-32
Optimum	28
pH range for growth	6.0-8.0
Optimum	7.0
NaCl range for growth (%)	0-8
Optimum	4
Utilization as a sole carbon source:	
Glucose	+
Maltose	+
Butyrate	+
Fructose	+
Sucrose	+
Acetate	+
Formate	+
Starch	+
Isomaltose	+
Trehalose	+
Galactose	−
Cellulose	−
Xylose	−
Lactate	+
Ethanol	+
D-mannose	+
Glycerin	+
Rhamnose	+
Sorbitol	−
DNA G+C content (mol%)	38.21 %
Isolation source	deep-sea sediments

273 **Supplementary Table 5. Statistical analysis the genes associated with DNA**
274 **degradation in Xianfuyuplasma coldseepsis zrk13 and other related strains.**

**Strain name**	**Query Seq-id**	**Subject Seq-id**	**Identical matches (%)**	**E-value**	**Bit score**
***Bacillus cellulosilyticus* DSM 2522**	Exonuclease	ADU30490.1	23.196	3.12E-12	61.6
	Exonuclease	ADU30296.1	30.061	7.24E-12	61.6
	Exonuclease	ADU30392.1	26.437	5.43E-08	49.7
	Exonuclease	ADU30276.1	26.347	2.89E-07	47.4
	Deoxyribonuclease	ADU28331.1	28.906	3.95E-35	123
	Exodeoxyribonuclease	ADU29978.1	38.307	1.76E-101	308
	Endoribonuclease	ADU32390.1	29.921	3.49E-09	52.4
	Bifunctional oligoribonuclease and PAP phosphatase NrnA	ADU31475.1	36.333	6.62E-29	107
	Purine nucleoside phosphorylase	ADU29815.1	36.471	9.15E-15	66.6
	Adenosine deaminase	ADU28313.1	44.792	1.35E-10	52
	Pyrimidine-nucleoside phosphorylase	ADU30053.1	40.509	4.30E-38	137
	Adenine deaminase	ADU30269.1	34.99	5.74E-35	133
***Bacillus cereus* ATCC 14579**	Exonuclease	QCX95386.1	88.172	0	511
	Exonuclease	QCX96690.1	28.571	8.37E-12	60.5
	Exonuclease	QCX95657.1	22.46	9.15E-06	43.9
	HNH endonuclease domain	QCX93051.1	41.168	6.56E-153	483
	HNH endonuclease domain	QCX93051.1	34.375	1.10E-16	82.8
	Deoxyribonuclease	QCX92163.1	33.977	8.23E-44	146
	Deoxyribonuclease	QCX92324.1	25.292	3.27E-23	92.4
	Exodeoxyribonuclease	QCX96011.1	36.818	1.52E-101	308

	Bifunctional oligoribonuclease and PAP phosphatase NrnA	QCX96423.1	41.522	2.43E-29	108
	Purine nucleoside phosphorylase	QCX93411.1	33.333	4.59E-12	59.7
	Adenosine deaminase	QCX92146.1	40.351	6.74E-08	45.1
	Pyrimidine-nucleoside phosphorylase	QCX93757.1	46.305	3.06E-45	156
	Pyrimidine-nucleoside phosphorylase	QCX95917.1	46.305	1.75E-44	154
Bacillus krulwichiae **AM31D**	Exonuclease	ARK30423.1	24.737	1.34E-15	70.9
	Exonuclease	ARK30883.1	29.545	8.78E-13	63.9
	Exonuclease	ARK32820.1	26.471	4.89E-06	43.5
	Deoxyribonuclease	ARK28434.1	31.923	4.26E-41	139
	Deoxyribonuclease	ARK31368.1	21.028	9.62E-10	54.3
	Exodeoxyribonuclease	ARK31393.1	38.063	1.76E-113	338
	Extracellular 'Staphylococcus nuclease homologue' - thermonuclease	ARK31241.1	43.704	4.42E-32	114
	Bifunctional oligoribonuclease and PAP phosphatase NrnA	ARK31710.1	35.235	5.10E-25	97.1
	Purine nucleoside phosphorylase	ARK31202.1	30.89	6.85E-11	56.2
	Adenosine deaminase	ARK28413.1	37.963	2.52E-10	51.2
	Pyrimidine-nucleoside phosphorylase	ARK29495.1	41.687	2.54E-40	143
	Pyrimidine-nucleoside phosphorylase	ARK29068.1	42.857	1.19E-35	130
	Cytidine deaminase	ARK29334.1	32.609	1.42E-09	50.1

	Cytidine deaminase	ARK29072.1	31.731	7.74E-06	39.7
	Adenine deaminase	ARK28714.1	30.058	1.57E-30	120
	Guanine deaminase	ARK28413.1	38.554	6.16E-06	40
***Bacillus lentus* NCTC4824**	Exonuclease	ARK30423.1	24.737	1.34E-15	70.9
	Exonuclease	ARK30883.1	29.545	8.78E-13	63.9
	Exonuclease	ARK32820.1	26.471	4.89E-06	43.5
	Deoxyribonuclease	ARK28434.1	31.923	4.26E-41	139
	Deoxyribonuclease	ARK31368.1	21.028	9.62E-10	54.3
	Exodeoxyribonuclease	ARK31393.1	38.063	1.76E-113	338
	Extracellular 'Staphylococcus nuclease homologue' - thermonuclease	ARK31241.1	43.704	4.42E-32	114
	Bifunctional oligoribonuclease and PAP phosphatase NrnA	ARK31710.1	35.235	5.10E-25	97.1
	Purine nucleoside phosphorylase	ARK31202.1	30.89	6.85E-11	56.2
	Adenosine deaminase	ARK28413.1	37.963	2.52E-10	51.2
	Pyrimidine-nucleoside phosphorylase	ARK29495.1	41.687	2.54E-40	143
	Pyrimidine-nucleoside phosphorylase	ARK29068.1	42.857	1.19E-35	130
	Adenine deaminase	ARK28714.1	30.058	1.57E-30	120
	Guanine deaminase	ARK28413.1	38.554	6.16E-06	40
***Bacillus megaterium* NBRC 15308 = ATCC 14581**	Exonuclease	SQI60196.1	29.24	1.35E-08	52.4
	HNH endonuclease domain	SQI54136.1	35.404	1.36E-15	75.9
	Deoxyribonuclease	SQI51120.1	31.25	2.57E-42	142
	Deoxyribonuclease	SQI53210.1	27.863	2.05E-26	100
	Exodeoxyribonuclease	SQI56226.1	38.702	1.16E-103	313

	Extracellular ribonuclease Bsn	SQI54136.1	34.568	2.14E-14	68.6
	Extracellular 'Staphylococcus nuclease homologue' - thermonuclease	SQI60725.1	42.308	6.93E-30	111
	Bifunctional oligoribonuclease and PAP phosphatase NrnA	SQI54963.1	40.878	8.71E-36	125
	Purine nucleoside phosphorylase	SQI56952.1	33.508	4.00E-13	62.4
	Cytidine deaminase	AJI23489.1	31.193	4.67E-08	46.2
	Cytidine deaminase	AJI25023.1	28.205	8.47E-06	40
	Pyrimidine-nucleoside phosphorylase	SQI53174.1	43.842	5.37E-46	158
***Bacillus weihaiensis* Alg07**	Exonuclease	APH05455.1	25.749	1.50E-09	53.9
	Exonuclease	APH04804.1	25.434	2.01E-08	50.4
	Exonuclease	APH06936.1	26.282	2.76E-06	43.9
	Exonuclease	APH05223.1	26.316	8.05E-06	43.9
	HNH endonuclease domain	APH03814.1	62.222	1.23E-92	302
	HNH endonuclease domain	APH03317.1	36.641	4.21E-35	143
	HNH endonuclease domain	APH03317.1	28.947	2.91E-07	52
	Deoxyribonuclease	APH06737.1	32.422	1.75E-44	147
	Exodeoxyribonuclease	APH04457.1	38.942	4.21E-105	317
	Extracellular ribonuclease Bsn	APH03814.1	71.239	5.21E-122	350
	Bifunctional oligoribonuclease and PAP phosphatase NrnA	APH04095.1	39.322	2.56E-38	132
	Purine nucleoside phosphorylase	APH04728.1	32.768	9.94E-11	55.5
	Adenosine deaminase	APH06755.1	42.202	3.46E-08	45.4
	Pyrimidine-nucleoside	APH06996.1	44.784	7.80E-42	147

	phosphorylase				
	Cytidine deaminase	APH04343.1	27.523	2.05E-06	41.6
	Adenine deaminase	APH05621.1	32.172	4.08E-35	134
***Alkaliphilus metalliredigens* QYMF**	Exonuclease	ABR48667.1	32.298	4.83E-13	63.5
	Exonuclease	ABR48830.1	30.588	2.24E-11	61.2
	Deoxyribonuclease	ABR46353.1	30.98	1.55E-40	137
	Exodeoxyribonuclease	ABR48656.1	35.632	7.84E-93	284
	Endoribonuclease	ABR48473.1	30.303	3.21E-07	47
	5'-nucleotidase	ABR48788.1	30.38	1.36E-06	44.3
	Purine nucleoside phosphorylase	ABR50308.1	33.484	1.65E-11	57.8
	Adenosine deaminase	ABR46269.1	37.398	2.77E-06	40.4
	Pyrimidine-nucleoside phosphorylase	ABR48680.1	43.005	1.43E-38	139
	Adenine deaminase	ABR50638.1	39.171	1.03E-57	196
***Clostridium cellulovorans* 743B**	Exonuclease	ADL53968.1	26.289	1.42E-15	71.6
	Exonuclease	ADL51527.1	30.769	2.29E-10	58.2
	HNH endonuclease domain	ADL53944.1	64	1.61E-95	311
	HNH endonuclease domain	ADL52314.1	56.25	1.24E-16	82.4
	HNH endonuclease domain	ADL52314.1	34	3.66E-10	61.6
	HNH endonuclease domain	ADL52879.1	42.857	1.87E-15	78.6
	HNH endonuclease domain	ADL52879.1	35.632	1.11E-07	53.5
	HNH endonuclease domain	ADL52500.1	41.379	6.47E-10	60.8
	HNH endonuclease domain	ADL50748.1	46.667	1.44E-09	60.1
	HNH endonuclease domain	ADL50748.1	43.478	1.55E-08	56.6
	Deoxyribonuclease	ADL50102.1	35.271	9.65E-50	161
	Exodeoxyribonuclease	ADL51678.1	37.356	9.08E-83	258
	Extracellular ribonuclease Bsn	ADL53944.1	79.039	1.50E-142	404

	Purine nucleoside phosphorylase	ADL50678.1	35.065	1.88E-14	66.2
	Pyrimidine-nucleoside phosphorylase	ADL51785.1	41.469	5.78E-40	142
***Clostridium diolis* DSM 15410**	Exonuclease	QES75040.1	28.571	2.30E-16	73.9
	Exonuclease	QES74293.1	28.022	4.51E-06	45.1
	Exonuclease	QES71798.1	28.824	4.92E-06	44.3
	Deoxyribonuclease	QES71356.1	31.356	1.70E-41	140
	Exodeoxyribonuclease	QES72847.1	34.783	3.94E-87	269
	Endoribonuclease	QES72543.1	25.229	1.27E-08	53.1
	Endoribonuclease	QES73113.1	38.889	1.11E-07	48.5
	Endoribonuclease	QES71790.1	26.357	5.22E-07	46.6
	Purine nucleoside phosphorylase	QES74206.1	35.586	3.88E-18	76.3
	Purine nucleoside phosphorylase	QES75170.1	33.478	7.83E-15	67.4
	Adenosine deaminase	QES76005.1	49.296	3.90E-08	45.8
	Cytidine deaminase	QES73804.1	37.209	4.01E-25	90.5
	Cytidine deaminase	QES74209.1	27.941	3.38E-10	52
	Adenine deaminase	QES72549.1	33.136	1.16E-28	115
	Xanthine dehydrogenase	QES71770.1	35.269	5.22E-39	148
***Clostridium ljungdahlii* DSM 13528**	Exonuclease	ADK16117.1	28.022	7.56E-07	47
	Exonuclease	ADK16682.1	25.294	7.92E-06	43.5
	Deoxyribonuclease	ADK13264.1	30.709	5.52E-36	125
	Deoxyribonuclease	ADK13946.1	22	2.13E-18	79
	Exodeoxyribonuclease	ADK14181.1	32.037	4.32E-88	271
	Endoribonuclease	ADK17061.1	31.25	1.65E-08	50.4
	Endoribonuclease	ADK13812.1	29.008	1.20E-06	45.1

	5'-nucleotidase	ADK16515.1	38.462	1.13E-06	44.3
	Cytidine deaminase	ADK14875.1	30.769	5.92E-09	48.5
	Pyrimidine-nucleoside phosphorylase	ADK14879.1	43.596	5.51E-40	142
	Adenine deaminase	ADK15452.1	32.707	1.27E-30	120
	Adenine deaminase	ADK16038.1	28.361	2.91E-20	90.1
	Xanthine dehydrogenase	ADK16042.1	35.269	2.35E-43	160
	Xanthine dehydrogenase	ADK15448.1	33.824	1.21E-38	147
***Desulfosporosinus orientis* DSM 765**	Exonuclease	AET66395.1	32.24	9.83E-17	75.5
	Exonuclease	AET69658.1	28.571	5.44E-06	44.7
	Deoxyribonuclease	AET65798.1	32	3.84E-40	137
	Exodeoxyribonuclease	AET66716.1	41.781	5.97E-101	305
	Adenosine deaminase	AET65775.1	36.036	1.01E-07	44.7
	Pyrimidine-nucleoside phosphorylase	AET66742.1	39.523	1.11E-32	123
	Adenine deaminase	AET67047.1	30.297	5.08E-24	101
	Xanthine dehydrogenase	AET69892.1	32.331	2.91E-36	140
***Absiella dolichum* DSM 3991**	Deoxyribonuclease	EDP11771.1	37.109	9.15E-37	144
	Exodeoxyribonuclease	EDP11206.1	37.709	3.08E-68	250
	Extracellular 'Staphylococcus nuclease homologue' - thermonuclease	EDP12353.1	43.448	9.17E-26	108
	Purine nucleoside phosphorylase	EDP10588.1	44.56	2.77E-28	116
	Adenosine deaminase	EDP11649.1	37.273	3.54E-06	43.1
	Pyrimidine-nucleoside phosphorylase	EDP11167.1	38.889	1.60E-36	145
	Guanine deaminase	EDP11649.1	44.444	9.50E-07	45.2

***Bulleidia extructa* W1219**	Deoxyribonuclease	EFC06429.1	30.916	1.87E-31	112
	Exodeoxyribonuclease	EFC05126.1	32.751	2.34E-71	229
	Purine nucleoside phosphorylase	EFC05958.1	41.15	1.36E-25	94.4
	Cytidine deaminase	EFC05232.1	31.405	8.47E-08	43.9
***Erysipelatoclostridium ramosum* DSM 1402**	HNH endonuclease domain	EDS18575.1	25.798	6.39E-12	67
	HNH endonuclease domain	EDS18575.1	33.858	2.46E-08	55.5
	HNH endonuclease domain	EDS18575.1	32.394	9.03E-06	47
	Deoxyribonuclease	EDS19578.1	31.496	6.86E-43	143
	Exodeoxyribonuclease	EDS17701.1	34.793	1.49E-93	286
	Extracellular 'Staphylococcus nuclease homologue' - thermonuclease	EDS20188.1	37.594	6.16E-24	90.5
	Bifunctional oligoribonuclease and PAP phosphatase NrnA	EDS17628.1	30	3.45E-11	58.2
	Purine nucleoside phosphorylase	EDS18798.1	36.957	2.85E-18	75.9
	Pyrimidine-nucleoside phosphorylase	EDS19190.1	41.089	9.51E-39	139
	Cytidine deaminase	EDS19163.1	34.286	6.95E-09	48.1
***Erysipelothrix larvae* LV19**	Deoxyribonuclease	AMC92460.1	33.2	6.00E-38	130
	Exodeoxyribonuclease	AMC93331.1	29.89	3.79E-65	213
	Extracellular 'Staphylococcus nuclease homologue' - thermonuclease	AMC92952.1	35.606	3.55E-20	80.5
	Purine nucleoside phosphorylase	AMC93921.1	41.558	2.60E-29	104
	Purine nucleoside	AMC93274.1	30.89	3.95E-08	47.8

	phosphorylase				
	Pyrimidine-nucleoside phosphorylase	AMC93394.1	39.151	5.71E-35	128
	Cytidine deaminase	AMC93367.1	27.586	5.65E-07	42.4
	Adenine deaminase	AMC93824.1	26.984	1.23E-21	93.6
***Faecalibaculum rodentium* ALO17**	Exonuclease	AMK55242.1	28	5.73E-07	45.1
	Deoxyribonuclease	AMK55887.1	27.953	2.24E-27	102
	Exodeoxyribonuclease	AMK54370.1	34.943	9.42E-81	254
	Endoribonuclease	AMK55505.1	29.688	7.01E-07	45.1
	Purine nucleoside phosphorylase	AMK53265.1	34.348	1.69E-18	76.3
	Adenosine deaminase	AMK53145.1	38.889	7.74E-06	38.9
	Adenine deaminase	AMK54993.1	28.18	6.67E-23	97.4
***Faecalibacillus faecis* SNUG30370**	Deoxyribonuclease	PST41358.1	28.346	2.03E-39	134
	Exodeoxyribonuclease	PST41762.1	34.211	6.12E-78	246
	Endoribonuclease	PST41237.1	23.256	3.22E-06	43.1
	Endoribonuclease	PST36747.1	29.921	4.43E-06	42.7
	Bifunctional oligoribonuclease and PAP phosphatase NrnA	PST41607.1	27.667	3.10E-09	52
	Purine nucleoside phosphorylase	PST39406.1	36.818	2.09E-18	75.9
	Pyrimidine-nucleoside phosphorylase	PST42037.1	41.327	5.93E-33	122
***Turicibacter sanguinis* PC909**	Deoxyribonuclease	EFF62507.1	31.25	9.60E-41	137
	Exodeoxyribonuclease	EFF62886.1	35.817	1.47E-94	289
	Endoribonuclease	EFF64252.1	29.493	2.91E-12	63.2
	Endoribonuclease	EFF63951.1	44.231	5.02E-08	48.5
	Bifunctional oligoribonuclease	EFF63265.1	32.323	1.56E-21	87

	and PAP phosphatase NrnA				
	Bifunctional oligoribonuclease and PAP phosphatase NrnA	EFF63284.1	30.69	4.75E-20	83.2
	5'-nucleotidase	EFF63266.1	29.018	6.79E-09	50.1
	Purine nucleoside phosphorylase	EFF63908.1	39.13	6.02E-20	80.1
	Adenosine deaminase	EFF62654.1	42.708	4.03E-10	50.4
	Pyrimidine-nucleoside phosphorylase	EFF63921.1	56.12	1.40E-73	230
	Cytidine deaminase	EFF63573.1	32.624	3.02E-22	82.4
	Cytidine deaminase	EFF63100.1	29.71	1.43E-10	52.4
	Adenine deaminase	EFF62948.1	30.476	4.39E-27	110
	Adenine deaminase	EFF64180.1	27.152	5.04E-11	60.8
	Guanine deaminase	EFF63642.1	44.34	2.00E-12	56.6
***Haloplasma contractile* SSD-17B**	Exonuclease	ERJ12906.1	27.835	2.48E-09	54.3
	HNH endonuclease domain	ERJ13593.1	26.57	2.05E-08	55.5
	Deoxyribonuclease	ERJ13349.1	28.682	3.20E-36	126
	Exodeoxyribonuclease	ERJ13776.1	35.44	3.41E-89	275
	Endoribonuclease	ERJ12547.1	29.134	1.36E-07	47.4
	Endoribonuclease	ERJ12547.1	32.857	3.16E-06	43.5
	Extracellular 'Staphylococcus nuclease homologue' - thermonuclease	ERJ13592.1	35.354	1.64E-12	62
	Extracellular 'Staphylococcus nuclease homologue' - thermonuclease	ERJ13594.1	27.879	2.30E-09	52.8
	Bifunctional oligoribonuclease and PAP phosphatase NrnA	ERJ13600.1	33.444	4.93E-19	80.5

	Bifunctional oligoribonuclease and PAP phosphatase NrnA	ERJ12830.1	30.717	6.49E-14	66.2
	5'-nucleotidase	ERJ13555.1	34.468	1.49E-17	74.3
	Purine nucleoside phosphorylase	ERJ13569.1	41.048	2.97E-23	89
	Adenosine deaminase	ERJ12601.1	40	3.01E-07	43.1
	Pyrimidine-nucleoside phosphorylase	ERJ12826.1	42.857	1.80E-41	146
	Cytidine deaminase	ERJ12215.1	30	5.82E-16	66.2
	Cytidine deaminase	ERJ13822.1	32.258	7.79E-11	53.1
	Adenine deaminase	ERJ11699.1	30.642	3.65E-22	95.5
***Candidatus* Izemoplasma sp. HR1**	HNH endonuclease domain	AIO18892.1	51.22	2.72E-73	244
	HNH endonuclease domain	AIO18361.1	37.705	3.55E-56	197
	Deoxyribonuclease	AIO18033.1	31.395	2.93E-42	141
	Exodeoxyribonuclease	AIO19333.1	34.437	1.95E-84	262
	Extracellular ribonuclease Bsn	AIO18892.1	48.507	3.64E-80	241
	Extracellular ribonuclease Bsn	AIO18361.1	42.294	3.85E-57	185
	Extracellular 'Staphylococcus nuclease homologue' - thermonuclease	AIO18242.1	30.37	2.20E-14	67
	Bifunctional oligoribonuclease and PAP phosphatase NrnA	AIO18237.1	27.517	2.20E-11	58.2
	Bifunctional oligoribonuclease and PAP phosphatase NrnA	AIO18238.1	28.912	2.61E-10	55.1
	5'-nucleotidase	AIO18167.1	30.093	8.90E-15	65.9
	Purine nucleoside phosphorylase	AIO19149.1	44.828	8.00E-34	116
	Pyrimidine-nucleoside	AIO19161.1	43.781	4.60E-41	144

	phosphorylase				
	Cytidine deaminase	AIO19003.1	30.631	4.96E-10	50.4
***Candidatus* Izemoplasma sp. HR2**	Exonuclease	KFZ26654.1	27.811	2.37E-07	45.8
	HNH endonuclease domain	KFZ27274.1	49.206	8.47E-71	240
	HNH endonuclease domain	KFZ26092.1	34.583	1.69E-29	121
	Deoxyribonuclease	KFZ26954.1	30.709	3.51E-40	135
	Exodeoxyribonuclease	KFZ26168.1	34.247	1.11E-84	263
	Extracellular ribonuclease Bsn	KFZ27274.1	49.064	1.64E-79	243
	Extracellular ribonuclease Bsn	KFZ26092.1	39.394	9.90E-39	139
	Endoribonuclease	KFZ27029.1	40.741	1.48E-07	47
	Extracellular 'Staphylococcus nuclease homologue' - thermonuclease	KFZ26564.1	33.803	9.08E-18	77
	Extracellular 'Staphylococcus nuclease homologue' - thermonuclease	KFZ26566.1	26.144	3.41E-06	42.7
	5'-nucleotidase	KFZ26880.1	27.778	7.18E-10	52.4
	Purine nucleoside phosphorylase	KFZ25539.1	49.351	1.04E-39	131
	Adenosine deaminase	KFZ25750.1	36.893	2.17E-07	42.4
	Pyrimidine-nucleoside phosphorylase	KFZ25647.1	44.75	6.39E-44	152
	Cytidine deaminase	KFZ26432.1	27.928	2.83E-09	48.5
	Adenine deaminase	KFZ26798.1	42.509	1.31E-63	211
	Guanine deaminase	KFZ26801.1	48.182	3.43E-15	63.5
	Guanine deaminase	KFZ25750.1	42.169	9.42E-07	41.2
	Xanthine dehydrogenase	KFZ26795.1	43.433	1.65E-77	254
Candidatus	HNH endonuclease domain	PAT02264.1	50.36	3.44E-80	267

**Izemoplasma sp. ZiA1**	HNH endonuclease domain	PAT01372.1	39.7	8.60E-49	175
	HNH endonuclease domain	PAT00971.1	41.126	1.38E-36	144
	HNH endonuclease domain	PAT02601.1	33.544	7.34E-11	62.8
	Deoxyribonuclease	PAT02457.1	31.349	1.37E-44	147
	Exodeoxyribonuclease	PAT01085.1	32.048	1.20E-82	257
	Extracellular ribonuclease Bsn	PAT02264.1	60	3.62E-92	275
	Extracellular ribonuclease Bsn	PAT01372.1	42.966	4.59E-56	181
	Extracellular ribonuclease Bsn	PAT00971.1	40.343	1.43E-40	144
	Extracellular 'Staphylococcus nuclease homologue' - thermonuclease	PAT02602.1	32.593	4.36E-14	66.2
	5'-nucleotidase	PAT02539.1	30.233	1.97E-14	65.1
	Purine nucleoside phosphorylase	PAT01849.1	46.753	5.96E-37	124
	Pyrimidine-nucleoside phosphorylase	PAT01838.1	44.444	1.04E-45	157
	Cytidine deaminase	PAT02361.1	28.889	6.28E-12	55.5
	Adenine deaminase	PAT01582.1	40.698	5.65E-62	206
	Guanine deaminase	PAT01577.1	48.598	4.75E-15	63.2
	Xanthine dehydrogenase	PAT01584.1	41.716	3.83E-79	258
***Xianfuyuplasma coldseepsis* zrk 13**	Exonuclease	G4Z02_00890	24.121	4.18E-07	46.2
	Exonuclease	G4Z02_07310	25.822	1.11E-06	45.4
	HNH endonuclease domain	G4Z02_00115	57.456	2.51E-77	256
	HNH endonuclease domain	G4Z02_00245	40.714	1.17E-54	192
	HNH endonuclease domain	G4Z02_04160	42.857	1.92E-33	136
	HNH endonuclease domain	G4Z02_00205	47.273	2.87E-31	129
	Deoxyribonuclease	G4Z02_06320	30.315	4.65E-39	132
	Exodeoxyribonuclease	G4Z02_07995	35.648	2.19E-94	288

	Extracellular ribonuclease Bsn	G4Z02_00115	58.333	2.39E-84	253
	Extracellular ribonuclease Bsn	G4Z02_00245	40.364	2.24E-58	187
	Extracellular 'Staphylococcus nuclease homologue' - thermonuclease	G4Z02_04155	33.835	1.29E-18	79.3
	5'-nucleotidase	G4Z02_04575	31.05	1.81E-13	62.4
	Purine nucleoside phosphorylase	G4Z02_08765	49.315	2.62E-35	120
	Adenosine deaminase	G4Z02_04405	36.667	4.40E-06	38.9
	Pyrimidine-nucleoside phosphorylase	G4Z02_08700	43.488	1.13E-34	127
	Cytidine deaminase	G4Z02_09160	29.524	6.04E-09	47.4
	Adenine deaminase	G4Z02_01130	41.276	2.89E-57	194
	Guanine deaminase	G4Z02_01105	49.495	7.55E-15	62.8
	Xanthine dehydrogenase	G4Z02_01145	40.476	3.85E-66	223
***Acholeplasma axanthum* NCTC10138**	Exonuclease	VEU80677.1	28.646	7.05E-09	50.8
	Exonuclease	VEU80275.1	26.882	1.29E-08	51.6
	HNH endonuclease domain	VEU80128.1	36.681	5.93E-35	135
	HNH endonuclease domain	VEU80129.1	33.987	1.06E-33	131
	HNH endonuclease domain	VEU80131.1	35.102	8.47E-27	113
	HNH endonuclease domain	VEU80127.1	38.415	6.55E-20	90.1
	HNH endonuclease domain	VEU80053.1	29.197	3.01E-07	50.1
	Deoxyribonuclease	VEU80210.1	33.203	5.60E-49	158
	Exodeoxyribonuclease	VEU80610.1	29.932	4.33E-73	233
	Extracellular ribonuclease Bsn	VEU80129.1	38.605	8.03E-34	122
	Extracellular ribonuclease Bsn	VEU80131.1	34.008	4.53E-32	120
	Extracellular ribonuclease Bsn	VEU80128.1	35.398	2.24E-31	116

	Extracellular ribonuclease Bsn	VEU80127.1	37.725	1.40E-17	77.8
	Bifunctional oligoribonuclease and PAP phosphatase NrnA	VEU80058.1	31.058	1.61E-18	78.6
	Bifunctional oligoribonuclease and PAP phosphatase NrnA	VEU80057.1	25.083	2.40E-08	49.3
	5'-nucleotidase	VEU80824.1	30	8.26E-12	58.2
	Purine nucleoside phosphorylase	VEU80820.1	41.739	1.81E-28	102
	Pyrimidine-nucleoside phosphorylase	VEU80812.1	43.784	2.14E-39	140
	Cytidine deaminase	VEU81004.1	31.818	4.36E-08	45.1
Acholeplasma brassicae **O502**	Exonuclease	CCV65533.1	28.814	2.89E-11	59.7
	HNH endonuclease domain	CCV66511.1	37.591	4.07E-43	156
	HNH endonuclease domain	CCV66163.1	38.667	8.92E-36	144
	HNH endonuclease domain	CCV66501.1	35.683	2.41E-34	133
	HNH endonuclease domain	CCV65582.1	31.293	1.89E-30	120
	HNH endonuclease domain	CCV66679.1	34.194	5.46E-15	74.7
	Deoxyribonuclease	CCV65333.1	33.597	8.27E-50	160
	Exodeoxyribonuclease	CCV66143.1	35.347	1.84E-92	283
	Extracellular ribonuclease Bsn	CCV66511.1	42.424	1.24E-46	154
	Extracellular ribonuclease Bsn	CCV66163.1	40	6.81E-42	149
	Extracellular ribonuclease Bsn	CCV65582.1	36.398	1.22E-33	121
	Extracellular ribonuclease Bsn	CCV66501.1	35.683	9.77E-33	120
	Extracellular 'Staphylococcus nuclease homologue' - thermonuclease	CCV66679.1	37.349	5.49E-08	48.1
	Bifunctional oligoribonuclease and PAP phosphatase NrnA	CCV66676.1	32.095	6.51E-16	71.2

	Bifunctional oligoribonuclease and PAP phosphatase NrnA	CCV66677.1	28.041	4.80E-12	60.1
	5'-nucleotidase	CCV65791.1	29.362	1.13E-09	52
	Purine nucleoside phosphorylase	CCV65795.1	41.81	6.15E-30	106
	Pyrimidine-nucleoside phosphorylase	CCV65804.1	42.356	3.93E-37	134
	Cytidine deaminase	CCV65734.1	30.909	2.30E-07	43.1
***Acholeplasma hippikon* NCTC10172**	HNH endonuclease domain	VEU82681.1	36.948	2.79E-32	126
	HNH endonuclease domain	VEU82948.1	36.325	1.12E-31	129
	HNH endonuclease domain	VEU83065.1	40.26	3.34E-25	103
	HNH endonuclease domain	VEU83049.1	28.125	1.23E-06	48.1
	Deoxyribonuclease	VEU82907.1	31.746	1.16E-47	154
	Exodeoxyribonuclease	VEU82176.1	34.091	3.10E-87	270
	Extracellular ribonuclease Bsn	VEU82948.1	39.565	1.73E-36	132
	Extracellular ribonuclease Bsn	VEU82681.1	33.74	7.79E-28	105
	Extracellular ribonuclease Bsn	VEU83065.1	37.662	1.26E-23	93.6
	Bifunctional oligoribonuclease and PAP phosphatase NrnA	VEU83051.1	31.12	2.65E-11	57.8
	Bifunctional oligoribonuclease and PAP phosphatase NrnA	VEU83050.1	28.041	7.14E-11	56.2
	5'-nucleotidase	VEU82383.1	26.16	1.86E-10	53.9
	Purine nucleoside phosphorylase	VEU82380.1	45.299	3.06E-34	117
	Pyrimidine-nucleoside phosphorylase	VEU82366.1	42.149	6.02E-32	119
	Cytidine deaminase	VEU82049.1	31.304	3.95E-08	45.1
Acholeplasma	Exonuclease	ABX81506.1	27.568	5.26E-06	42

***laidlawii* PG-8A**	HNH endonuclease domain	ABX80743.1	36.522	2.60E-32	131
	HNH endonuclease domain	ABX80786.1	34.926	1.23E-30	118
	HNH endonuclease domain	ABX81840.1	41.42	4.20E-27	115
	HNH endonuclease domain	ABX81840.1	32.7	4.43E-25	108
	HNH endonuclease domain	ABX80780.1	40	8.37E-26	105
	HNH endonuclease domain	ABX80781.1	30	1.96E-23	103
	HNH endonuclease domain	ABX81998.1	31.2	2.56E-06	47
	Deoxyribonuclease	ABX80815.1	29.762	3.63E-40	135
	Exodeoxyribonuclease	ABX81095.1	30.752	1.14E-73	234
	Extracellular ribonuclease Bsn	ABX80786.1	38.71	2.40E-35	123
	Extracellular ribonuclease Bsn	ABX80743.1	36.91	1.11E-31	119
	Extracellular ribonuclease Bsn	ABX80781.1	33.203	4.24E-24	97.4
	Extracellular ribonuclease Bsn	ABX80780.1	36.129	2.09E-22	90.5
	Bifunctional oligoribonuclease and PAP phosphatase NrnA	ABX81996.1	28.197	3.41E-15	68.9
	Bifunctional oligoribonuclease and PAP phosphatase NrnA	ABX81997.1	27.541	4.80E-13	62.8
	5'-nucleotidase	ABX81317.1	28.085	3.91E-09	50.1
	Purine nucleoside phosphorylase	ABX81314.1	44.397	4.48E-33	114
	Adenosine deaminase	ABX80701.1	39.241	2.04E-07	42.4
	Pyrimidine-nucleoside phosphorylase	ABX81299.1	44.133	8.93E-40	140
	Cytidine deaminase	ABX81559.1	32.143	4.57E-08	44.7
	Guanine deaminase	ABX80701.1	40.816	4.00E-06	38.9
Acholeplasma oculi	HNH endonuclease domain	CDR31368.1	45.509	1.19E-32	125
	HNH endonuclease domain	CDR31412.1	36.726	1.14E-30	125
	HNH endonuclease domain	CDR31373.1	34.263	7.64E-29	121

	HNH endonuclease domain	CDR31530.1	30	2.80E-09	56.6
	Deoxyribonuclease	CDR31350.1	29.741	1.04E-35	124
	Exodeoxyribonuclease	CDR30693.1	33.484	1.86E-83	260
	Extracellular ribonuclease Bsn	CDR31412.1	36.842	4.02E-32	120
	Extracellular ribonuclease Bsn	CDR31368.1	44.099	7.96E-32	115
	Extracellular ribonuclease Bsn	CDR31373.1	31.128	1.15E-24	99
	Bifunctional oligoribonuclease and PAP phosphatase NrnA	CDR31528.1	28.713	4.32E-14	65.9
	5'-nucleotidase	CDR30842.1	29.787	2.12E-09	50.8
	Purine nucleoside phosphorylase	CDR30839.1	44.397	4.71E-33	114
	Pyrimidine-nucleoside phosphorylase	CDR30827.1	42.119	8.91E-36	130
***Acholeplasma palmae* J233**	HNH endonuclease domain	CCV63642.1	36.364	3.42E-34	132
	HNH endonuclease domain	CCV64897.1	36.747	5.99E-23	101
	HNH endonuclease domain	CCV64995.1	31.646	3.36E-11	62.4
	Deoxyribonuclease	CCV64837.1	35.294	1.56E-50	162
	Exodeoxyribonuclease	CCV64651.1	32.28	1.70E-81	254
	Extracellular ribonuclease Bsn	CCV63642.1	35.622	2.49E-31	116
	Extracellular ribonuclease Bsn	CCV64897.1	33.473	5.54E-25	99.8
	Extracellular 'Staphylococcus nuclease homologue' - thermonuclease	CCV64995.1	34.314	7.74E-12	59.3
	Bifunctional oligoribonuclease and PAP phosphatase NrnA	CCV64993.1	34.256	2.25E-18	77.8
	Bifunctional oligoribonuclease and PAP phosphatase NrnA	CCV64994.1	25.817	8.65E-09	50.1
	5'-nucleotidase	CCV64447.1	27.35	5.98E-10	52.4

	Purine nucleoside phosphorylase	CCV64451.1	43.534	1.32E-32	112
	Pyrimidine-nucleoside phosphorylase	CCV64461.1	44.722	5.12E-37	133
	Cytidine deaminase	CCV64162.1	28.319	1.74E-08	45.8
***Mycoplasma anatis* NCTC10156**	HNH endonuclease domain	VEU73637.1	30.846	8.68E-16	75.5
	Deoxyribonuclease	VEU74149.1	24.906	4.12E-24	93.2
	Extracellular ribonuclease Bsn	VEU73637.1	32.099	4.99E-16	72
	Extracellular 'Staphylococcus nuclease homologue' - thermonuclease	VEU73989.1	29.762	3.84E-12	58.2
	Bifunctional oligoribonuclease and PAP phosphatase NrnA	VEU73681.1	23.986	1.27E-09	52
	Bifunctional oligoribonuclease and PAP phosphatase NrnA	VEU73677.1	22.145	1.75E-09	51.6
	Purine nucleoside phosphorylase	VEU73634.1	40	4.75E-22	84.7
	Pyrimidine-nucleoside phosphorylase	VEU73635.1	40.419	1.56E-28	109
	Cytidine deaminase	VEU73684.1	26.154	9.58E-06	38.1
***Mycoplasma bovis* PG45**	Exonuclease	ADR25071.1	26.23	7.10E-06	42
	HNH endonuclease domain	ADR25008.1	33.14	4.76E-12	63.9
	Deoxyribonuclease	ADR25272.1	25.483	9.72E-29	105
	Extracellular ribonuclease Bsn	ADR25008.1	29.762	1.18E-12	61.6
	Extracellular 'Staphylococcus nuclease homologue' - thermonuclease	ADR25351.1	34.211	6.14E-10	51.6
	Purine nucleoside	ADR25207.1	39.13	2.37E-23	87.8

	phosphorylase				
	Purine nucleoside phosphorylase	ADR25093.1	42.478	5.05E-11	54.7
	Pyrimidine-nucleoside phosphorylase	ADR24937.1	37.368	1.03E-28	110
***Mycoplasma citelli* NCTC10181**	HNH endonuclease domain	VEU74851.1	26.396	1.83E-20	92.4
	Deoxyribonuclease	VEU74756.1	29.545	1.43E-34	120
	Extracellular ribonuclease Bsn	VEU74851.1	28.485	3.91E-12	61.2
	Extracellular 'Staphylococcus nuclease homologue' - thermonuclease	VEU74492.1	29.94	4.97E-11	55.5
	Bifunctional oligoribonuclease and PAP phosphatase NrnA	VEU74520.1	24.407	3.09E-11	57.4
	Purine nucleoside phosphorylase	VEU74489.1	35.065	1.27E-18	75.9
	Pyrimidine-nucleoside phosphorylase	VEU74488.1	38.861	7.90E-35	127
***Mycoplasma dispar* ATCC 27140**	Deoxyribonuclease	AJR12464.1	25.283	1.46E-24	96.7
	Purine nucleoside phosphorylase	AJR12237.1	34.973	2.89E-10	52.8
	Pyrimidine-nucleoside phosphorylase	AJR12236.1	46.193	3.19E-20	85.9
***Mycoplasma gallinaceum* NCTC10183**	HNH endonuclease domain	VEU59023.1	31.66	3.98E-17	81.6
	Deoxyribonuclease	VEU58692.1	30.736	2.54E-27	101
	Extracellular ribonuclease Bsn	VEU59023.1	31.641	4.02E-16	73.2
	Extracellular 'Staphylococcus nuclease homologue' -	VEU58427.1	27.273	2.34E-10	52.8

	thermonuclease				
	Bifunctional oligoribonuclease and PAP phosphatase NrnA	VEU58968.1	24.573	2.55E-11	57.4
	Bifunctional oligoribonuclease and PAP phosphatase NrnA	VEU58969.1	22.848	5.38E-09	50.4
	Purine nucleoside phosphorylase	VEU58577.1	37.339	2.70E-20	80.1
	Pyrimidine-nucleoside phosphorylase	VEU58576.1	39.474	3.80E-33	122
	Cytidine deaminase	VEU59036.1	29.091	3.43E-06	40.4
***Mycoplasma iowae* 695**	Exonuclease	QHG90004.1	28.276	3.30E-06	43.5
	Deoxyribonuclease	QHG90219.1	25.94	3.81E-34	119
	Bifunctional oligoribonuclease and PAP phosphatase NrnA	QHG90009.1	29.878	9.32E-07	43.9
	Purine nucleoside phosphorylase	QHG90067.1	32.636	4.75E-14	63.5
	Pyrimidine-nucleoside phosphorylase	QHG90068.1	36.404	2.05E-14	68.9
	Cytidine deaminase	QHG90069.1	28.283	1.79E-08	45.8
***Mycoplasma leachii* PG50**	Deoxyribonuclease	ADR24065.1	23.954	1.82E-32	115
	Deoxyribonuclease	ADR24097.1	23.954	5.92E-24	92.8
	Exodeoxyribonuclease	ADR24666.1	29.004	2.28E-72	231
	Endoribonuclease	ADR23830.1	29.323	4.96E-07	44.7
	Purine nucleoside phosphorylase	ADR24157.1	31.319	6.02E-09	48.5
	Pyrimidine-nucleoside phosphorylase	ADR24604.1	36.935	1.15E-21	90.1
Mycoplasma	Deoxyribonuclease	VEU59555.1	23.438	6.34E-26	100

***neurolyticum* NCTC10166**	Extracellular 'Staphylococcus nuclease homologue' - thermonuclease	VEU59720.1	27.273	3.95E-08	47
	Purine nucleoside phosphorylase	VEU59368.1	34.634	9.55E-20	78.6
	Pyrimidine-nucleoside phosphorylase	VEU59367.1	41.504	8.77E-31	115
***Mycoplasma salivarium* NCTC10113**	HNH endonuclease domain	VEU56257.1	33.728	2.09E-11	63.2
	Deoxyribonuclease	VEU55617.1	23.954	3.20E-32	115
	Deoxyribonuclease	VEU54716.1	23.954	1.04E-23	92.8
	Deoxyribonuclease	VEU56589.1	22.692	1.62E-21	86.3
	Exodeoxyribonuclease	VEU54648.1	29.004	3.99E-72	231
	Extracellular ribonuclease Bsn	VEU56257.1	29.392	3.10E-12	61.6
	Endoribonuclease	VEU55550.1	29.323	8.71E-07	44.7
	Extracellular 'Staphylococcus nuclease homologue' - thermonuclease	VEU56372.1	29.353	8.13E-11	55.8
	Purine nucleoside phosphorylase	VEU56102.1	35.354	2.74E-17	72.8
	Purine nucleoside phosphorylase	VEU55819.1	31.319	1.06E-08	48.5
	Pyrimidine-nucleoside phosphorylase	VEU56101.1	35.45	2.96E-28	109
	Pyrimidine-nucleoside phosphorylase	VEU55584.1	36.935	2.02E-21	90.1
***Ureaplasma parvum* serovar 1 str. ATCC 27813**	Deoxyribonuclease	EDT48857.1	24.809	2.51E-24	93.2
	Purine nucleoside phosphorylase	EDT49121.1	36.986	8.68E-16	67.4

	Pyrimidine-nucleoside phosphorylase	EDT48986.1	41.558	9.74E-17	75.1
	Cytidine deaminase	EDT49004.1	29.545	1.59E-11	53.1
***Ureaplasma urealyticum* serovar 10 str. ATCC 33699**	Deoxyribonuclease	ACI59820.1	24.615	1.19E-25	97.1
	Purine nucleoside phosphorylase	ACI59969.1	36.986	2.52E-17	72
	Pyrimidine-nucleoside phosphorylase	ACI60103.1	41.558	8.37E-17	75.5
	Cytidine deaminase	ACI60252.1	29.545	2.99E-11	52.8
***Ureaplasma urealyticum* 4318**	Deoxyribonuclease	WP_038101569.1	24.71	2.10E-28	104
	Purine nucleoside phosphorylase	WP_038101860.1	33.19	2.49E-15	66.6
	Pyrimidine-nucleoside phosphorylase	WP_051749400.1	39.655	2.71E-17	77
	Cytidine deaminase	WP_038102595.1	29.31	5.34E-10	49.3
***Entomoplasma ellychniae* ELCN-1**	Deoxyribonuclease	PPE04836.1	25.373	6.45E-28	103
	Deoxyribonuclease	PPE04838.1	26.054	4.96E-25	95.5
	Exodeoxyribonuclease	PPE04563.1	27.828	2.24E-67	216
	Purine nucleoside phosphorylase	PPE04896.1	42.487	7.95E-19	75.9
	Pyrimidine-nucleoside phosphorylase	PPE04916.1	38.333	9.04E-21	87.4
	Cytidine deaminase	PPE04940.1	23.134	1.13E-07	43.1
***Entomoplasma freundtii* BARC 318**	Deoxyribonuclease	ATZ16064.1	28.736	1.10E-31	113
	Deoxyribonuclease	ATZ16065.1	25.66	5.45E-24	92.4
	Exodeoxyribonuclease	ATZ16337.1	32.045	8.58E-65	209
	Purine nucleoside phosphorylase	ATZ16502.1	40.69	6.41E-16	68.2

	Pyrimidine-nucleoside phosphorylase	ATZ16565.1	29.558	5.64E-16	73.2
***Entomoplasma luminosum* PIMN-1**	Deoxyribonuclease	ATZ16910.1	28.244	1.94E-35	123
	Deoxyribonuclease	ATZ16911.1	28.302	1.35E-25	97.1
	Exodeoxyribonuclease	ATZ17329.1	35.971	2.30E-65	211
	Endoribonuclease	ATZ17107.1	31.25	2.43E-09	51.2
	Purine nucleoside phosphorylase	ATZ17169.1	38.326	1.60E-23	88.6
	Pyrimidine-nucleoside phosphorylase	ATZ17497.1	33.816	2.88E-17	77
	Cytidine deaminase	ATZ17277.1	25.714	4.06E-06	39.3
***Entomoplasma melaleucae* M1**	Deoxyribonuclease	ATZ17656.1	29.771	6.34E-28	102
	Deoxyribonuclease	ATZ17655.1	21.673	4.57E-23	89.7
	Exodeoxyribonuclease	ATZ18018.1	34.539	1.16E-63	206
	Purine nucleoside phosphorylase	ATZ18366.1	39.151	3.44E-20	79.3
	Cytidine deaminase	ATZ17751.1	28.873	4.81E-11	52
***Entomoplasma somnilux* PYAN-1**	Deoxyribonuclease	ATZ18423.1	29.536	3.54E-36	124
	Deoxyribonuclease	ATZ18424.1	26.744	1.53E-27	102
	Exodeoxyribonuclease	ATZ18759.1	30.993	1.24E-75	238
	Endoribonuclease	ATZ18707.1	29.703	1.71E-06	42.7
	Bifunctional oligoribonuclease and PAP phosphatase NrnA	ATZ18560.1	30.07	6.40E-12	58.9
	Purine nucleoside phosphorylase	ATZ18413.1	37.826	4.61E-20	79.3
	Pyrimidine-nucleoside phosphorylase	ATZ18410.1	34.856	2.74E-26	103
	Cytidine deaminase	ATZ19014.1	25.185	1.21E-06	40.4

***Mesoplasma chauliocola* CHPA-2**	Deoxyribonuclease	ASZ08811.1	26.894	1.04E-34	120
	Deoxyribonuclease	ASZ08812.1	28.517	2.70E-25	95.9
	Exodeoxyribonuclease	ASZ09096.1	35.145	3.09E-58	192
	Purine nucleoside phosphorylase	ASZ09432.1	34.222	1.51E-18	75.1
	Pyrimidine-nucleoside phosphorylase	ASZ08879.1	36.364	1.25E-25	101
***Mesoplasma coleopterae* BARC 779**	HNH endonuclease domain	ATZ20542.1	39.914	2.17E-39	146
	Deoxyribonuclease	ATZ20574.1	24.242	2.36E-28	104
	Deoxyribonuclease	ATZ20575.1	28.788	4.15E-27	100
	Exodeoxyribonuclease	ATZ20859.1	33.882	3.54E-62	202
	Extracellular ribonuclease Bsn	ATZ20542.1	39.474	2.15E-43	147
	Purine nucleoside phosphorylase	ATZ21189.1	38.117	7.47E-19	75.9
	Pyrimidine-nucleoside phosphorylase	ATZ20647.1	37.437	1.05E-28	109
	Cytidine deaminase	ATZ20998.1	30.328	2.65E-13	58.2
	Cytidine deaminase	ATZ20671.1	24.638	2.34E-07	42.4
***Mesoplasma entomophilum* TAC**	Deoxyribonuclease	ATQ35212.1	28.244	7.07E-26	97.4
	Deoxyribonuclease	ATQ35211.1	23.106	1.38E-25	97.1
	Exodeoxyribonuclease	ATQ35493.1	33.882	1.16E-61	201
	Purine nucleoside phosphorylase	ATQ35834.1	38.117	2.17E-19	77.4
	Pyrimidine-nucleoside phosphorylase	ATQ35280.1	37.94	2.99E-28	108
	Cytidine deaminase	ATQ35644.1	29.508	1.08E-12	56.6
***Mesoplasma florum* L1**	Deoxyribonuclease	AAT75404.1	29.771	1.99E-29	107
	Deoxyribonuclease	AAT75403.1	24.242	4.58E-29	105

	Exodeoxyribonuclease	AAT75688.1	33.553	1.06E-60	198
	Purine nucleoside phosphorylase	AAT76021.1	39.462	1.09E-19	78.2
	Pyrimidine-nucleoside phosphorylase	AAT75475.1	38.191	5.06E-28	107
	Cytidine deaminase	AAT75824.1	30	1.38E-14	61.2
***Mesoplasma lactucae* ATCC 49193**	Deoxyribonuclease	ATG97711.1	25	4.02E-27	100
	Deoxyribonuclease	ATG97713.1	25.095	7.40E-23	89.4
	Exodeoxyribonuclease	ATG97391.1	31.223	4.18E-75	238
	Purine nucleoside phosphorylase	ATG97224.1	31.25	9.59E-12	56.6
***Mesoplasma syrphidae* YJS**	Deoxyribonuclease	AUF83222.1	26.996	3.75E-31	111
	Deoxyribonuclease	AUF83224.1	26.038	1.29E-23	91.7
	Bifunctional oligoribonuclease and PAP phosphatase NrnA	AUF83348.1	33.449	8.61E-15	67
	Purine nucleoside phosphorylase	AUF83849.1	46	9.89E-07	42.4
	Pyrimidine-nucleoside phosphorylase	AUF83855.1	34.656	9.82E-23	92.8
**Mesoplasma tabanidae BARC 857**	Deoxyribonuclease	ATZ21254.1	29.545	1.28E-27	102
	Deoxyribonuclease	ATZ21253.1	22.642	1.08E-26	99.8
	Exodeoxyribonuclease	ATZ21568.1	31.68	6.17E-62	202
	Purine nucleoside phosphorylase	ATZ21909.1	38.117	2.60E-20	79.7
	Pyrimidine-nucleoside phosphorylase	ATZ21330.1	37.688	6.12E-28	107
***Spiroplasma alleghenense* PLHS-1**	Deoxyribonuclease	AXK51817.1	26.217	4.66E-28	103
	Exodeoxyribonuclease	AXK51173.1	28.668	2.61E-67	217

	Purine nucleoside phosphorylase	AXK50771.1	37.379	1.96E-18	75.5
	Pyrimidine-nucleoside phosphorylase	AXK51276.1	36.318	5.56E-24	97.1
	Cytidine deaminase	AXK51101.1	25.664	1.19E-06	40.8
***Spiroplasma citri* R8-A2**	Deoxyribonuclease	APE75778.1	29.297	3.70E-39	132
	Exodeoxyribonuclease	APE74732.1	33.71	9.62E-86	265
	Endoribonuclease	APE74544.1	31.25	3.25E-08	48.5
	Bifunctional oligoribonuclease and PAP phosphatase NrnA	APE73976.1	26.78	1.17E-17	75.9
	Purine nucleoside phosphorylase	APE74493.1	39.367	1.55E-17	73.2
	Pyrimidine-nucleoside phosphorylase	APE74428.1	37.566	1.67E-30	115
***Spiroplasma clarkii* CN-5**	Deoxyribonuclease	ATX71678.1	26.894	4.99E-31	112
	Exodeoxyribonuclease	ATX70975.1	28.833	4.48E-64	209
	Endoribonuclease	ATX70912.1	29.688	5.15E-08	47.8
	Purine nucleoside phosphorylase	ATX71587.1	39.024	1.39E-16	70.5
	Pyrimidine-nucleoside phosphorylase	ATX70848.1	37.245	3.77E-26	103
***Spiroplasma eriocheiris* DSM 21848**	HNH endonuclease domain	AKM54673.1	28.137	4.56E-10	58.2
	Deoxyribonuclease	AKM54779.1	30.83	7.35E-41	137
	Exodeoxyribonuclease	AKM54120.1	34.615	2.86E-91	279
	Extracellular ribonuclease Bsn	AKM54673.1	25.781	4.55E-13	63.5
	Endoribonuclease	AKM54031.1	27.907	6.02E-10	53.1
	Bifunctional oligoribonuclease and PAP phosphatase NrnA	AKM53665.1	25.407	1.54E-13	63.9

	Purine nucleoside phosphorylase	AKM53913.1	36.126	4.72E-17	71.6
	Pyrimidine-nucleoside phosphorylase	AKM54040.1	37.778	3.40E-29	111
	Cytidine deaminase	AKM54369.1	37.143	1.53E-15	64.3
***Spiroplasma floricola* 23-6**	Deoxyribonuclease	AUB32180.1	27.308	3.54E-29	106
	Exodeoxyribonuclease	AUB31644.1	30.529	2.86E-72	230
	Endoribonuclease	AUB31565.1	30.597	6.17E-10	53.1
	Endoribonuclease	AUB31504.1	30.769	6.23E-09	50.1
	Purine nucleoside phosphorylase	AUB31149.1	35.749	5.90E-16	68.6
	Pyrimidine-nucleoside phosphorylase	AUB31851.1	34.3	5.03E-22	91.3
	Cytidine deaminase	AUB31834.1	27.273	7.30E-07	41.2
***Spiroplasma helicoides* TABS-2**	Deoxyribonuclease	AOG61101.1	26.437	1.83E-30	110
	Exodeoxyribonuclease	AOG60519.1	29.773	1.38E-75	238
	Bifunctional oligoribonuclease and PAP phosphatase NrnA	AOG60857.1	34.574	1.31E-11	58.2
	Purine nucleoside phosphorylase	AOG60053.1	37.104	7.00E-16	68.6
	Pyrimidine-nucleoside phosphorylase	AOG60644.1	34.831	2.59E-23	95.1
	Cytidine deaminase	AOG60624.1	27.007	1.30E-09	48.9
***Spiroplasma litorale* TN-1**	Deoxyribonuclease	AKX34727.1	25.191	1.53E-31	113
	Exodeoxyribonuclease	AKX34136.1	29.398	3.82E-71	228
	Endoribonuclease	AKX34501.1	30.769	8.36E-09	49.7
	Endoribonuclease	AKX34501.1	31.461	1.87E-06	43.1
	Purine nucleoside	AKX33741.1	32.836	3.89E-13	60.8

	phosphorylase				
	Pyrimidine-nucleoside phosphorylase	AKX33928.1	36.75	9.74E-29	110
***Spiroplasma phoeniceum* P40**	Deoxyribonuclease	AXF97163.1	29.297	1.08E-38	131
	Exodeoxyribonuclease	AXF96432.1	34.615	3.39E-89	274
	Endoribonuclease	AXF96815.1	29.921	8.44E-10	53.1
	Bifunctional oligoribonuclease and PAP phosphatase NrnA	AXF96127.1	26.28	5.76E-16	71.2
	Purine nucleoside phosphorylase	AXF96339.1	38.288	4.53E-16	69.3
	Pyrimidine-nucleoside phosphorylase	AXF95513.1	38.005	4.27E-33	122
	Cytidine deaminase	AXF96561.1	28.889	2.89E-09	48.1
***Spiroplasma turonicum* Tab4c**	Deoxyribonuclease	AKU80329.1	24.521	3.27E-28	104
	Exodeoxyribonuclease	AKU79730.1	26.889	1.70E-68	221
	Purine nucleoside phosphorylase	AKU79326.1	38	9.13E-15	65.5
	Purine nucleoside phosphorylase	AKU79649.1	44.211	1.50E-08	48.1
	Pyrimidine-nucleoside phosphorylase	AKU79505.1	34.988	8.07E-22	90.9
